# Increasing fragmentation of forest cover in Brazil’s Legal Amazon from 2001 to 2017

**DOI:** 10.1038/s41598-020-62591-x

**Published:** 2020-04-02

**Authors:** Bruno Montibeller, Alexander Kmoch, Holger Virro, Ülo Mander, Evelyn Uuemaa

**Affiliations:** Department of Geography, University of Tartu, Tartu, 51003 Estonia

**Keywords:** Forest ecology, Environmental impact

## Abstract

Persistent forest loss in the Brazilian Legal Amazon (BLA) is responsible for carbon emission, reduction of ecosystem services, and loss of biodiversity. Combining spatial data analysis with high spatial resolution data for forest cover and forest loss, we quantified the spatial and temporal patterns of forest dynamics in the BLA. We identified an alarming trend of increasing deforestation, with especially high rates in 2016 and 2017. Moreover, the creation of forest cover fragments is faster than ever due to decreasing size and dispersion of forest loss patches. From 2001 to 2017, the number of large forest loss patches decreased significantly, accompanied by a reduction in the size of these patches. Enforcement of field inspections and of initiatives to promote forest conservation will be required to stop this trend.

## Introduction

Since the 1970s, the Brazilian Legal Amazon (BLA), a region that includes nine states in the Amazon Basin, has been a stage for intense land use and rapid land cover change^1^, despite the forest’s important roles as a carbon sink^2,3^ and biodiversity hotspot^4^. Cattle farming and soybean production have been important drivers of this process^5^. The forest loss increases the region’s carbon emission^6^ and reduces its ability to provide ecosystem functions^7^. Furthermore, these environmental problems have intensified due to forest fragmentation and the edge effects^8,9^ caused by deforestation.

The annual deforestation rates decreased from 27.8 km^2^ in 2004 to 4.6 km^2^ in 2012^10^. Among the factors responsible for this reduction (e.g., changes in commodity prices, land prices, government policies), important roles were played by the Action Plan for the Prevention and Control of Deforestation in the Legal Amazon^11^ (PPCDAm) and the Soy Moratorium^12,13^, which were launched in 2004 and 2006, respectively. Based on data from the national deforestation monitoring system^10^ (PRODES) provided by Brazil’s Instituto Nacional de Pesquisas Espaciais, the PPCDAm and Soy Moratorium programs were able to reduce deforestation. Using satellite images, PRODES has been monitoring deforestation in primary forest in the BLA and providing annual reports since 1988^10^.

Although PRODES data is crucial for monitoring deforestation rates, it provides an incomplete picture of the dynamics of the deforestation process for the whole BLA. For instance, PRODES defines deforestation as complete removal of the primary forest (dense tropical forest), but excludes other forest types (i.e., secondary forest, savannah, shrubland) and other forest disturbance dynamics (e.g., illegal selective logging and fire), and it does not consider deforestation of areas smaller than 6.25 ha^10,14^.

The global high-resolution (30-m) Global Forest Change (GFC) data developed by Hansen *et al*.^15^ can enhance assessments of forest dynamics for the BLA. GFC maps the forest loss caused by any type of disturbance, including fire, logging, loss of secondary forest, and small-scale forest loss (to a minimum area of 0.09 ha). Although PRODES and GFC have significant methodological differences, including their definitions of deforestation and forest loss, the two datasets generally agree on the trends in deforestation rates in the BLA^16,17^.

Most studies that have used either GFC or PRODES data focused on the area of deforestation^14,18^ and either omitted the spatial and structural pattern of the remaining forest cover, or analyzed it separately^19,20^. The remaining forest cover has mostly been studied based on its fragmentation^8,21^ processes and its consequences, such as changes in forest fire susceptibility^22,23^, habitat loss^24^, and carbon emission^6^. Brinck *et al*.^6^ calculated that 19% of the remaining tropical forest area was within 100 m of a forest edge, and that these edge areas were responsible for 31% of the estimated annual carbon emission from tropical deforestation. Using the GFC forest cover map for 2000, Taubert *et al*.^19^ developed models that predicted a high increase of forest fragmentation despite low rates of forest loss. They suggested that only the combination of a strong reduction of deforestation rates with increased reforestation efforts would decrease the fragmentation process. Moreover, Seymor and Harris^25^ propose that in order to reduce deforestation rates it is necessary to organize and apply policies that are designed accordingly to the specifications of each region, since the drivers of deforestation are complex and are under continuous changes.

Even though several studies have provided information on either forest loss or forest fragmentation, we found no long-term, high-resolution, wall-to-wall studies that combine analysis of forest loss and forest fragmentation in the BLA. Given the abovementioned limitations and the potential to improve our understanding of forest dynamics in the Amazon Basin, we designed the present study to provide a more complete understanding of the changes in forest loss and forest cover fragmentation in the BLA. The questions used as support to design the study were: (i) how did the trends in forest loss influence the forest fragmentation process? (ii) did the forest fragmentation increase or decrease over time? (iii) was forest fragmentation different in primary and secondary forest? We hypothesized that despite the decrease of the forest loss rates, the forest fragmentation rates did not decrease and that would be related to the spatial changes of the forest loss process. We defined forest loss as areas of significant disturbance (i.e., fire degradation, selective logging) or total removal of the tree cover canopy^15^, and defined forest cover as areas (Landsat pixels) with >30% tree cover^14,19,26^. The forest cover analysis were divided into areas of primary forest and non-primary forest. The primary forest areas were defined using the PRODES data of primary forest as a mask. Since we used PRODES primary forest data as a mask, we defined primary forest areas as disturbed and undisturbed old-growth humid tropical forest within the tropical forest biome^27,28^. The term “non-primary” forest that we adopted includes the remaining area of forest cover outside of the PRODES primary forest mask, but within the BLA. On this basis, our definition of non-primary forest includes mainly secondary rainforest areas (forest that regrew after a disturbance), savannas (Cerrado), shrublands, dry broadleaf forests, and Pantanal flooded savannas. Unfortunately, there is insufficient data available on yearly forest regrowth, as these forests take time to regrow. We also made the simplifying assumption that forest regrowth after disturbance (secondary forest) in the BLA would not mitigate the fragmentation problem, as regenerating forest is not a target of Brazilian forest policy; these forests are being cut and converted to other land uses or cover types on an ongoing basis^29,30^, and these changes have intensified since 2010^31^.

The objectives of our work were to evaluate the spatio-temporal pattern of forest loss and forest cover fragmentation from 2001 to 2017. We separately examined areas within and outside of indigenous reserves and conservation units where the forest is either managed under more strict regulations (i.e., sustainable use) or where forest is completely protected (i.e., integral protection conservation units and indigenous reserves)^32^, and separately examined primary and non-primary forest. We used spatial landscape metrics and hotspot analysis to capture the spatial and temporal patterns of change in forest loss and forest cover throughout the BLA by focusing on whether the processes differ between the primary and non-primary forest types and between the areas inside and outside of the indigenous reserves and conservation areas.

## Results

### Spatial and temporal patterns of forest loss

**Figure 1:**
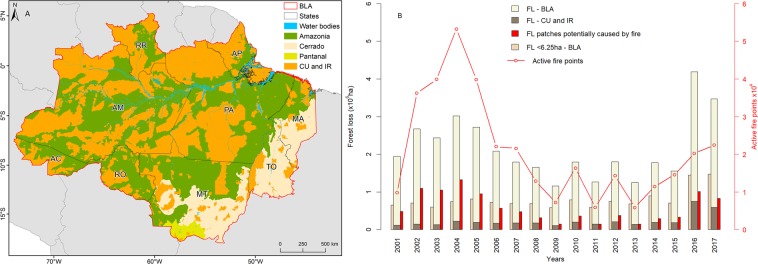
Forest loss within the Brazilian Legal Amazon (BLA). (**A**) Map of the study area. Amazonia is the biome composed mainly by moist broadleaf forest, Cerrado is the biome composed of tropical savanna and grasslands, and Pantanal is the biome flooded during the rainy season and that supports trees and shrubs. (**B**) annual forest loss (FL) area within and outside conservation units (CU) and indigenous reserves (IR), as well as the area occupied by forest loss patches smaller than 6.25 ha in the BLA and the number of active fire points within the forest loss patches and the forest loss area potentially caused by fire. Brazilian states: Acre (AC), Amazonas (AM), Amapá (AP), Maranhão (MA), Mato Grosso (MT), Pará (PA), Rondônia (RO), Roraima (RR), and Tocantins (TO).

From 2001 to 2017, 36.6 × 10^6^ ha of forest either suffered disturbance or was removed. The total forest loss differed among years (Fig. 1, Table S1). From 2001 to 2004, forest loss rates increased rapidly (+54.0%). After the 2004 peak, forest loss rates decreased, with some fluctuations, until 2015 (–48.3%). The rate increased greatly in 2016 and 2017. The number of patches influenced by fire and the forest loss area potentially caused by fire closely followed these trends. In 2004, 44.0% of the total forest loss area (1.3 × 10^6^ ha) showed at least one active fire point, versus 22% (0.33 × 10^6^ ha) in 2015. The area of forest loss patches potentially caused by fire also decreased, from 76.8 ha in 2004 to 42.2 ha in 2015, but then increased to 115.0 and 74.9 ha in 2016 and 2017, respectively.

From 2001 to 2017, more than 3.8 × 10^6^ ha (Table S1) of forest loss (10.6% of the total loss) took place within conservation units and indigenous reserves (Fig. 1A), at an average rate >196 × 10^3^ ha year^−1^ and with the greatest value in 2016, at 751 × 10^3^ ha. Of the total forest loss (3.8 × 10^6^ ha), 9% was within integral protection units, 53% was within sustainable use areas, 36% was within indigenous reserves, and ~2% was in areas with overlaps between the conservation units and indigenous reserves (Table S2).

**Figure 2:**
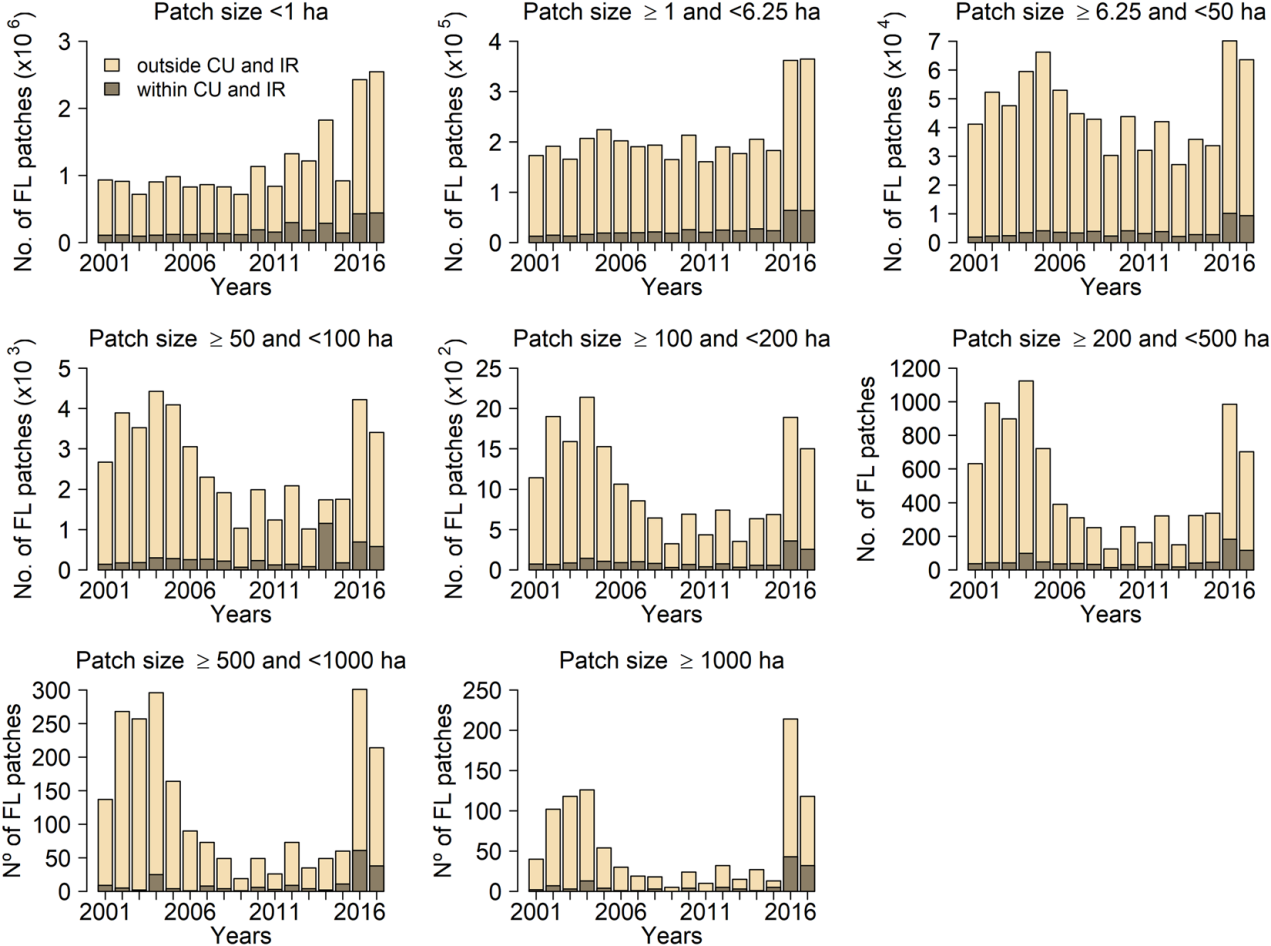
Annual forest loss patch size distribution. Changes in the number of forest loss patches in each size class from 2001 to 2017 outside and within conservation units (CU) and indigenous reserves (IR). The sum of these categories equals the total number of forest loss patches in the BLA.

The area covered by small (<6.25 ha) forest loss patches, as a proportion of the total forest loss, increased from 2001 to 2015; this is because the total annual forest loss decreased, but the area of small patches remained the same (Fig. 1B). The number of forest loss patches smaller than 1 ha, which are not considered in PRODES and hence are not reflected in the official Brazilian deforestation statistics, had increasing trend across all years and particularly in 2016 and 2017 (Fig. 2). Therefore, the areal reduction in forest loss from 2004 to 2015 resulted primarily from the decrease in the number of large forest loss patches (patches >6.25 ha). However, in 2016 and 2017, number of all forest loss patches increased considerably.

The number of very small (<1 ha) forest loss patches increased significantly (*P* < 0.05) both inside and outside conservation units and indigenous reserves (Fig. 2; Table S3). From 2004 (the first year of the PPCDAm program) to 2015, the number of large forest loss patches (≥6.25 ha) decreased by 45.2% (Fig. 2). However, during the same years, the number of forest loss patches ≥1 ha and <6.25 ha decreased by 11.0% and the number of patches smaller than 1 ha increased by 1.7%. In 2016 and 2017, which differed from most other years, the number of forest loss patches increased compared to previous years.

**Figure 3:**
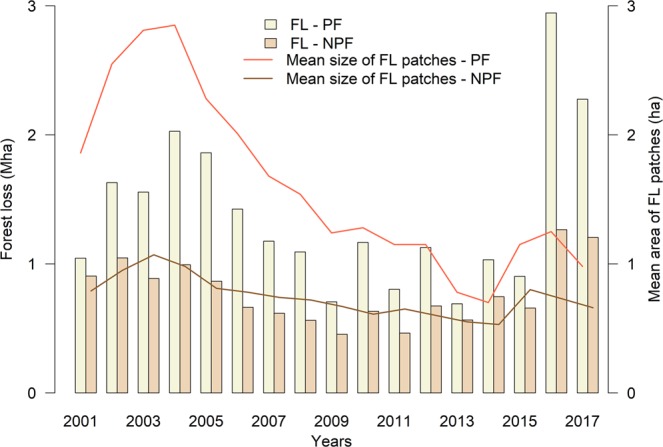
Annual forest loss for different forest types. Total forest loss (FL) and mean patch size of forest loss patches in primary forest (PF) and non-primary forest (NPF).

We also found that 23.4 × 10^6^ ha of forest loss (7% of the total area of primary forest in 2000) occurred within primary forest areas, versus 13.2 × 10^6^ ha in non-primary forest areas (17% of the total non-primary forest in 2000) (Fig. 3; Table S4). Forest loss decreased significantly in both primary and non-primary forest from 2001 until 2015, and then increased in 2016 and 2017 (Fig. 3). However, when we included forest loss in 2016 and 2017, then there was no significant trend (Table S4).

**Figure 4:**
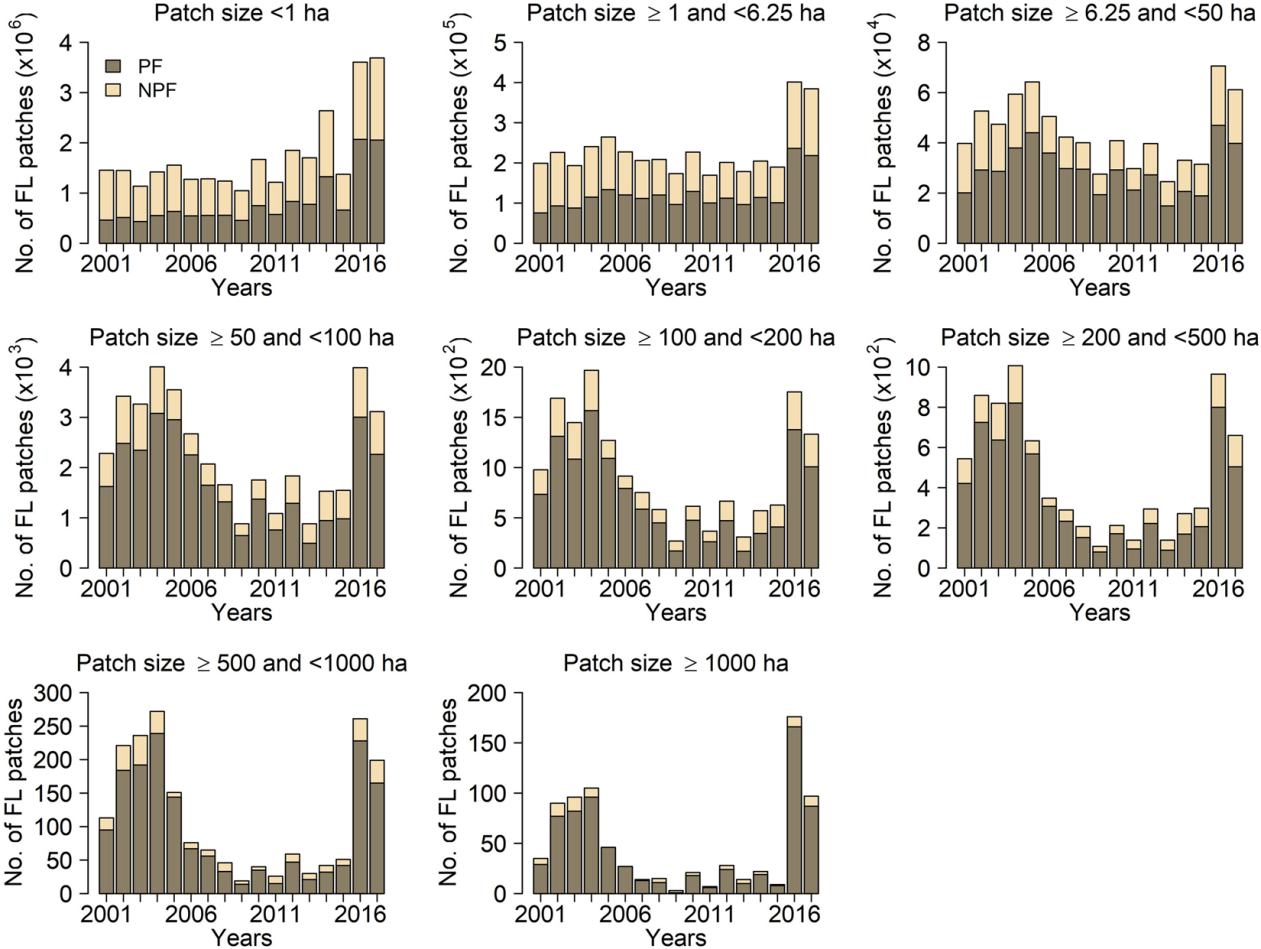
Annual forest loss patch size distribution for different forest types. The number of forest loss (FL) patches in each size class from 2001 to 2017 for primary (PF) and non-primary forest (NPF).

The mean size of forest loss patches decreased significantly (*P* < 0.05) in both forest types, mainly due to the decreased number of large forest loss patches (>6.25 ha) since 2004 to 2006 (Figs. 3 and 4; Table S5). In general, primary forest had more forest loss patches and proportionally more large forest loss patches than non-primary forest. The number of patches smaller than 6.25 ha did not decrease over the years, but increased after 2015.

**Figure 5:**
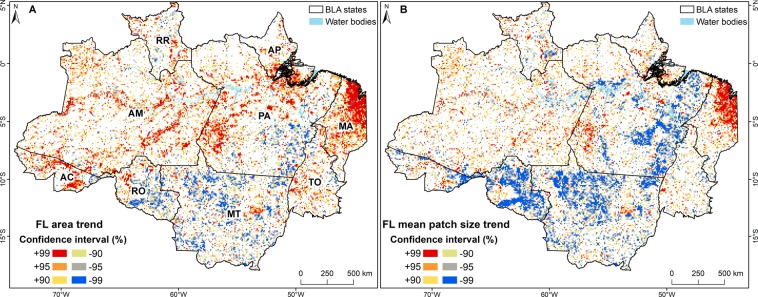
Spatial patterns of forest loss and mean patch size temporal trends in the Brazilian Legal Amazon (BLA). (**A**) Forest loss (FL) area trend and (**B**) mean patch size trend from 2001 to 2017. The confidence levels for the trends were estimated using the *Z* values. For the confidence intervals (CIs), *z* ≥ 2.576 for the 99% CI, 2.576 > *z* ≥ 1.960 for the 95% CI, and 1.960 > *z* ≥ 1.645 for the 90% CI. *Brazilian states: Acre (AC), Amazonas (AM), Amapá (AP), Maranhão (MA), Mato Grosso (MT), Pará (PA), Roraima (RR), Rondônia (RO), and Tocantins (TO)*.

The spatial distribution of statistically significant forest loss trends showed a clear pattern of increasing forest loss in Amazonas, Pará, Acre, and Maranhão states, which lie outside of the so-called “arc of deforestation” (Fig. 5A). The arc of deforestation is an area that extends from Maranhão to Acre and was the main agriculture frontier during the early 20 00s^33^. In total, 20.5% of the 10 km  ×  10 km fishnet grid cells showed a trend of increasing forest loss. Only 10.0% of the cells showed decreasing forest loss, and these were mainly located in the former deforestation areas, where most of the forest loss had occurred before the study period. The main regions of decreasing mean size of forest loss patches coincided with the areas where the forest loss was increasing (Fig. 5B). In total, 17.0% of the cells showed decreasing patch size (Fig. 5B).

We hypothesized that forest loss would occur close to areas where it occurred in previous years. Our analysis of forest loss adjacencies showed the highest spatial adjacency within 2 to 3 years of the initial forest loss (see Supplementary Fig. S1). For instance, 24.6% and 23.7% of the forest loss cells in 2007 were adjacent to cells that experienced forest loss in 2006 and 2005, respectively, meaning that forest loss in 2007 was more likely to occur adjacent to cells that experienced forest loss in 2006 and 2005 than in cells adjacent to cells that experienced forest loss in 2001.

**Figure 6:**
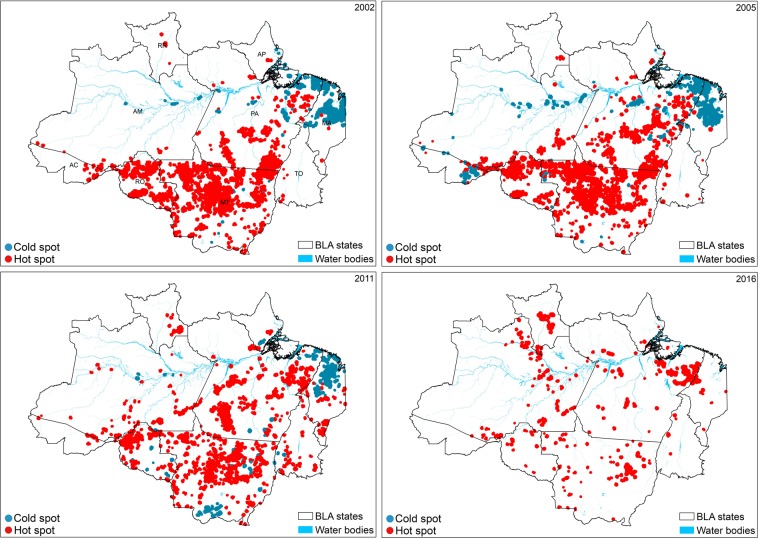
Spatial hotspot analysis of forest loss in the Brazilian Legal Amazon (BLA). Forest loss hot spots (clusters of large forest loss patches) and cold spots (clusters of small forest loss patches) in 2002 (before the Action Plan for the Prevention and Control of Deforestation in the Legal Amazon [PPCDAm] was implemented), 2005 (after PPCDAm but before the Soy Moratorium), and in 2011 and 2016 (when both programs were active). Only spots with a confidence interval ≥90% are shown.

Hot spot analysis showed that the forest loss hot spots (spatially aggregated large forest loss patches) were more concentrated in the central, southern, and southwestern parts of the BLA until 2006, whereas cold spots (spatially aggregated small forest loss patches) were present primarily in the northeast (Fig. 6). However, after 2006, the number of hot spots decreased and their location gradually shifted towards the northern part of the study area. An increase in the number of cold spots was observed from 2005 to 2011, especially in Maranhão state. The spread of the cold spots was also evident along the Amazon River and its tributaries, which are known to be important logging transportation routes. The appearance of the cold spots also means that the mean size of the forest loss patches decreased. The cold spots remained present until 2015, but there were no cold spots in 2016 and 2017.

### Forest cover fragmentation

**Figure 7:**
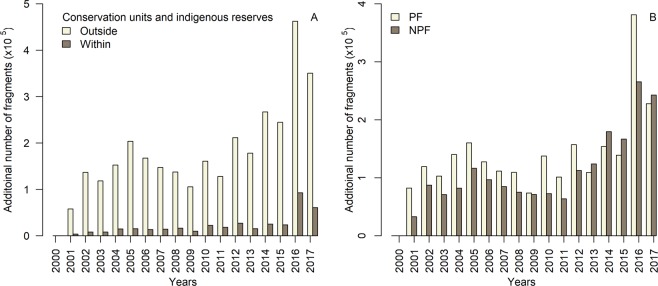
Annual dynamics of forest fragmentation in the Brazilian Legal Amazon. Changes in the number of forest cover fragments (**A**) within and outside conservation units (CU) and indigenous reserves (IR) and (**B**) for primary forest (PF) and non-primary forest (NPF).

In 2000, the BLA had 407 × 10^6^ ha of forest cover divided into >5.2 × 10^6^ fragments (Table S6). However, by 2017, the number of fragments had increased by 3.6 million (+68.5%). The mean patch size of forest cover fragments decreased from 77.5 ha in 2000 to 41.8 ha in 2017 (−46.1%; Table S6). The rate of fragmentation (addition of new fragments) has clearly been increasing since about 2010 despite generally lower rates of forest loss, with exceptionally high values in 2016 and 2017. At the same time, the edge density of the remaining forest cover is increasing (Table S6). The area of forest cover outside conservation units and indigenous reserves was 198 × 10^6^ ha in 2000, and was present as 4.4 million fragments. By 2017, the number of fragments had increased by 72.7% (to 7.6 million fragments). Conservation units and indigenous reserves had 208 × 10^6^ ha of forest in 2000, and was present as 800 000 fragments, and by 2017, the forest cover had decreased to 204 × 10^6^ ha (<0.1%), and was present as 1.2 million fragments (Tables S6 and S7; Fig. 7A). Separately, integral protection conservation units presented a decrease of 1% in the area of forest cover and an increase of 21.3% in the number of forest fragments. At the same time, sustainable use conservation units and indigenous reserves presented a decrease of 3.2% and 1.6% in the forest cover area and an increase of 51.6% and 46.8% in the number of forest fragments, respectively (Table S8).

By examining the fragmentation separately for primary and non-primary forest, we found that in 2000, the non-primary forest area of 77.5 × 10^6^ ha was divided into 6.2 × 10^6^ fragments, versus 329.5 × 10^6^ ha for the primary forest, which was divided into only 0.5 × 10^6^ fragments (Table S9). However, Fig. 7B shows that by 2017, the number of fragments had increased to 8.15 × 10^6^ for non-primary forest (+31.5%) and to 2.9 × 10^6^ for primary forest (+480.0%). This indicates that the forest cover fragmentation has been faster in primary forest than in non-primary forest. The addition of the new fragments has been increasing continuously since 2010, with exceptionally high values in 2016 and 2017.

The distribution of additional forest cover fragments in primary and non-primary forest generally increased for small patches (<50 ha). The number of primary forest patches smaller than 50 ha (0.5 × 10^6^ fragments) increased by 448.0% (2.9 × 10^6^ fragments), versus 32% for non-primary forest during the study period (Table S10). However, for forest cover patches larger than 50 ha, the number of non-primary forest patches decreased by 6.4%, versus a 42.0% decrease for primary forest patches (Table S10).

**Figure 8:**
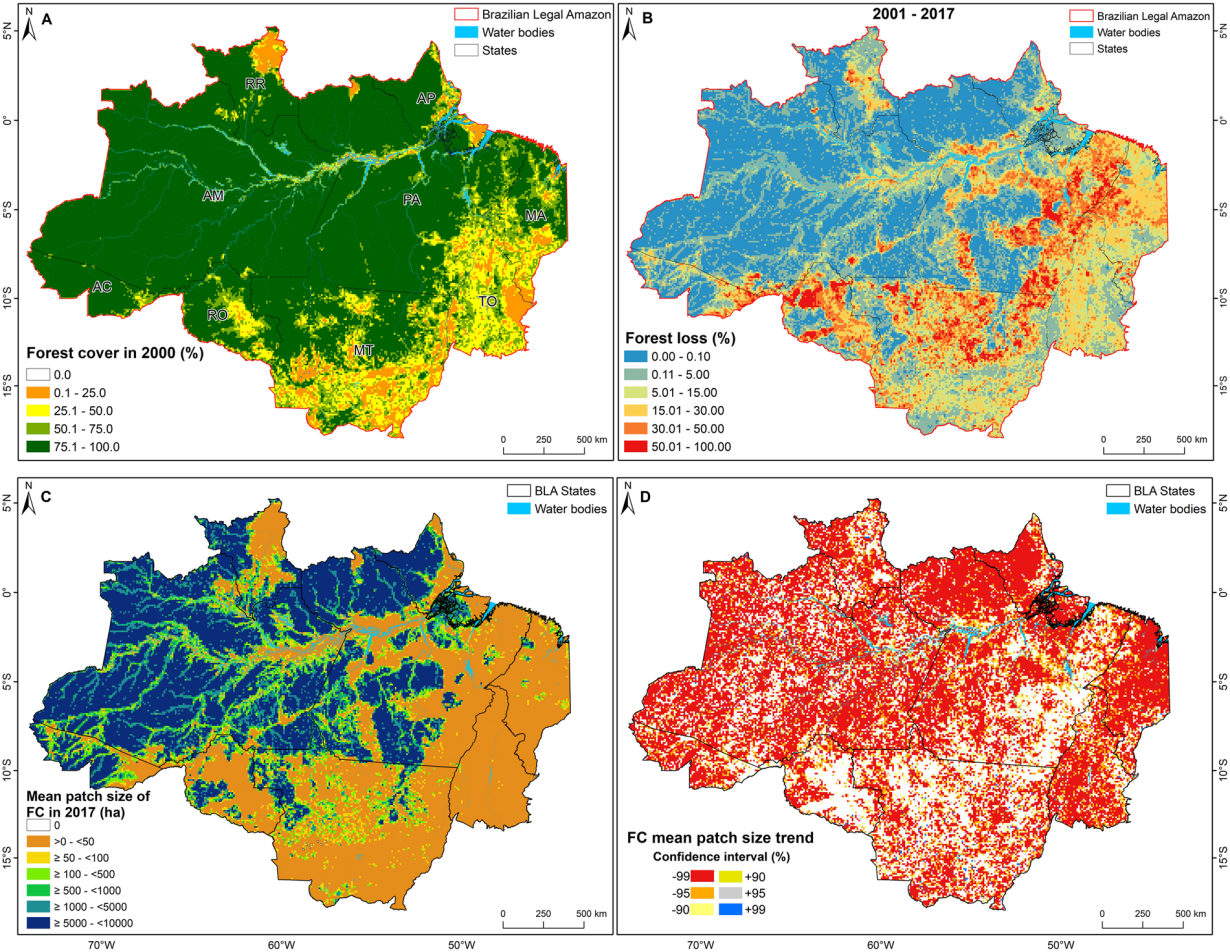
Forest dynamics in the Brazilian Legal Amazon (BLA). (**A**) Percentage forest cover (FC) in each 10 km  ×  10 km grid cell in 2000; (**B**) proportion of the forest cover affected by forest loss from 2001 to 2017, (**C**) mean patch size of cells with forest cover in 2017, and (**D**) statistically significant changes (Mann–Kendall test) in the mean forest cover patch size from 2001 to 2017. Brazilian states: Acre (AC), Amazonas (AM), Amapá (AP), Maranhão (MA), Mato Grosso (MT), Pará (PA), Roraima (RR), Rondônia (RO), and Tocantins (TO).

The fishnet grid analysis showed that in 2000, 69.6% of the grid cells had more than 75.0% forest cover (Fig. 8A), but that this had decreased to 58.5% by 2017(see Supplementary Fig. S2), largely due to large decreases in forests in this category in the northern, eastern, and southern parts of the study area, as well as along the rivers. In 2000, 19.0% of the grid cells had less than 50.0% forest cover. By 2017, 40.5% of the grid cells had lost more than 5.0% of their forest cover (Fig. 8B). The mean patch size of the remaining forest cover fragments has also been decreasing across the BLA. In 2017, almost half of the grid cells with forest cover (40.6%) had a mean patch size smaller than 50 ha (Fig. 8C), and trend analysis indicated that 68.7% of the grid cells had a decreasing mean patch size from 2000 to 2017 (Fig. 8D). Moreover, the spatial pattern of changes in forest cover clearly followed the small forest loss patches along the tributaries of the Amazon and revealed the spread of the deforestation pattern.

## Discussion

### Spatial and temporal patterns of forest loss

Although the PRODES and GFC methodologies differ, our analysis of forest loss in the BLA agrees with the analysis of Richards *et al*.^16^: both datasets reveal a similar temporal pattern, especially in terms of the known decrease of forest loss from 2004 until 2012, followed by gradually increasing deforestation^16^. As reported by previous studies, the decrease of forest loss mainly resulted from implementation of PPCDAm^34^ and the Soy Moratorium^13^. The reduction in the number of large forest loss patches, which was the target of these initiatives, contributed to this decrease^18^. However, there was an obvious increase in the number of small forest loss patches (<6.25 ha), and after 2008 these patches represented more than 47.0% of the total forest loss area in each year. The decrease of the mean forest loss patch size may indicate adoption of a strategy by landholders^35^ to avoid detection by the deforestation monitoring system^16,18^. Richards *et al*.^16^ reported that in the northern Mato Grosso and northeastern Pará states, landholders have learned how to avoid detection of forest loss. This finding was reinforced by our analysis, which showed that from 2008 to 2013, the forest loss patches not only became smaller in these regions, but also spread out more, which is what we would predict to occur if landowners were attempting to escape the monitoring. An additional explanation for this pattern is the increased contribution of small landholders to forest loss that was identified by Godar *et al*.^36^. They found that from 2005 to 2011, the annual rates of deforestation in smallholder areas increased by 69%. It is also important to note that the GFC product considers any forest disturbance to be forest loss, therefore any areas that were affected by selective logging and fire may contribute to the number of small forest loss patches. The occurrence of fire in the BLA is usually related to its use during the deforestation process or for agricultural and pasture management^37^. In some cases, especially during drought periods, fires can escape the site being managed and become forest fires. Our results showed that the fire occurrence pattern followed the forest loss pattern, indicating that partially the forest loss might have been caused by fire. Moreover, the general decrease in the number of active fire points in the BLA can be explained by a change from extensive to intensive land use methods (e.g., intensive agriculture) since intensive agriculture does not use fire deliberately^38^. The recent increase in fire occurrence can partially explain the forest loss in 2016 and 2017 (24.0% in 2016 and 23.9% in 2017). This is especially true in 2016, a year with an intense drought^39,40^. Studies have shown that intense droughts occur as a result from changes in the precipitation regime in the Amazon region^41,42^ due to anomalies in sea surface temperatures, and can potentially increase fire occurrence^43^ resulting in forest loss. In addition, changes in the Brazilian environmental legislation, for example the inclusion of riparian preservation areas in the calculation of the legal reserves, since 2012^44^, may have influenced the recent increase in forest loss^45–47^.

Even though the primary and non-primary forest loss results showed no significant trends when we included data from 2016 and 2017, a more evident reduction was observed for loss of primary forest, which was the focus of PPCDAm and the Soy Moratorium^12,13^ (Fig. 3). This resulted in a smaller difference between the total loss of primary and non-primary forest, especially after 2007 and 2008. Our spatial analysis using the fishnet grid approach to calculate landscape metrics as well as our hot spot analysis also revealed spatial and temporal changes in the dynamics of forest loss in both forest types. The change in the locations of forest loss from the states of Mato Grosso, Pará, and Rondônia from 2001 to 2007 (Fig. 6) to inner areas of primary forest in the states of Amazonas, Acre, and Roraima indicates that forest loss is now occurring in areas that were previously considered isolated from this process^48^ (mainly in Amazonas). This change was also followed by a decrease in the connectivity of the forest loss patches, which became more dispersed after 2008 (See supplementary Fig. S1), and their mean patch size increased in these new areas of forest loss compared with the old areas of forest loss. Significant new areas of forest loss in Maranhão and Tocantins states are also examples of this spatial change, especially within the areas of non-primary forest (i.e. the Cerrado biome). The latter pattern is related to the hypothesis of cross-biome leakage, which consists of the displacement of soybean cultivation and deforestation to the Cerrado as a result of the implementation of the Soy Moratorium^49,50^.

The relatively low and constant forest loss rates within conservation units and indigenous reserves from 2001 to 2015, followed by increases in 2016 and 2017, appear to indicate the efficiency of forest conservation in these areas. However, the smaller mean size of forest loss patches observed in these areas presents another challenge for the monitoring systems and conservation efforts. As Cabral *et al*.^32^ also noted, our results suggest that most of the forest loss occurred in areas with a low level of protection, such as environmental protection areas. It is important to note that we did not evaluate whether the protection level provided in different types of conservation unit affected the forest loss process.

### Increasing rate of forest fragmentation

The addition of new forest fragments slowed in 2004. However, the trend reversed in 2010, and forest fragmentation rates started to increase, reaching their highest levels in 2016 and 2017. This corroborates the results of Taubert *et al*.^19^, who used simulations to confirm that forest fragmentation will increase with a small additional amount of forest loss^19^. The forest cover fragmentation within conservation units and indigenous reserves was less intense than outside of these areas, with integral protection conservation units showing the lowest rate of fragmentation. This result agrees with the findings of Cabral *et al*.^32^, who stated that these areas actively contribute to improved conservation and to reducing or preventing forest fragmentation and degradation processes in the BLA. Outside of conservation units and indigenous reserves, the fragmentation increased and remained high even in years with decreased forest loss (e.g., 2011). A similar pattern of increasing forest cover fragmentation was observed for both primary and non-primary forests as well as in years with a low rate of forest loss. However, the fragmentation process was more intense for non-primary forest, as the number of new fragments exceeded the number of new primary forest fragments after 2013, even though the non-primary forest area is much smaller than the primary forest area. The increased fragmentation of non-primary forest, which are not considered in anti-deforestation initiatives (e.g. PPCDAm and Soy Moratorium), might be a result of the recent increase of non-primary forest loss observed by Tyukavina *et al*.^27^ and in our study. It is complicated to explain the forest cover fragmentation process and the drivers behind it because different fragmentation patterns result from the different agents who are responsible for the forest loss (e.g., miners, farmers, loggers)^51^. Our analysis also showed that the mean size of the remaining forest cover patches is decreasing over time, and this, combined with the increasing number of fragments, will have strong negative impacts on biodiversity^24,52^, edge effects^8^, carbon emission^6^, and susceptibility to fire^53^. Negative effects on biodiversity come from a reduction in the connectivity of the fragments^52^. According to percolation theory, increasing the number of forest fragments leads to a critical point for percolation at which the forest patches lose the connectivity^19^ that is essential for species migration^54^.

The change in the locations of forest loss from the traditional “arc of deforestation” region towards inner regions of the BLA and the Cerrado biome, combined with the reduction of the size of forest loss patches, explains the widespread and significant reduction of the mean size of forest cover patches and the increase of fragmentation in these regions. This trend may increase carbon emission because, according to Malhi *et al*.^55^ and Aragão *et al*.^56^, the western forest of the BLA has a higher net primary production than the eastern forest and, consequently, can assimilate more atmospheric carbon as biomass. Moreover, the increase of the edge density caused by increasing fragmentation is accompanied by a loss of biomass at forest edges^9^.

One possibility to reduce forest loss and consequently carbon emission would be to promote the preservation of secondary forest cover, which we classified as one type of non-primary forest in the present study. During the initial years of regrowth, these secondary forests show rapid biomass accumulation as well as high extraction of soil nutrients^57^, and therefore represent an important option to reduce forest fragmentation and its environmental impacts. Unfortunately, previous studies^29,49^ showed that these areas are undergoing ongoing cutting and regrowth, or are being converted to other land uses such as agriculture or pasture^31^. Moreover, these areas are also more susceptible to fire than primary forest^53,58^.

Spatial analysis showed how enforcement of conservation laws are changing the dynamics of forest loss and forest cover patterns in the BLA area. Our results illustrate the complex relationships between these two elements of the landscape and the challenges that remain to preserve the positive effects of Brazil’s forest conservation initiatives. One key result of our study is that it revealed the increase of small-scale forest loss and of forest cover fragmentation. Reducing the minimum mapping area threshold of the current Brazilian monitoring systems (e.g. PRODES or near real-time deforestation detection-DETER^59^) would be an important response to the new pattern of forest loss and improve the annual estimates of forest loss in the Amazon region. However, this reduction would not result in a decrease of forest loss if the government provides insufficient financial and human resources to support field inspections and to punish the responsible. Moreover, it is important to consider the objectives of the monitoring systems, since the reduction of the minimum mapping area for instance, would be costly and would compromise comparisons of the monitoring time series. In addition, the observed increase in forest loss in 2016 and 2017 is disturbing given the current situation in Brazil, in which the government shows no intention to promote forest conservation^52^. In future research, we recommend that researchers provide a more complete picture of the situation by including forest regrowth data in their analysis as new approaches for mapping areas with regrowth are being developed^60^. We also suggest that researchers combine data on the driving forces behind fragmentation with forest loss data to identify the types and the causes of forest fragmentation over time in different areas of the BLA (e.g. arc of deforestation and inner areas of the BLA) so that solutions can be developed.

## Methods

### Amazon forest cover and loss data

To assess the forest cover fragmentation and patterns of forest loss in the BLA, we used Version 1.5 of the 30-m-resolution GFC data developed by Hansen *et al*.^15^ for tree cover and forest loss. The tree *cover* data is the percentage (0‒100%) of tree cover in 2000 for each 30 m × 30 m cell. The forest *loss* data represents areas of significant disturbance (e.g., fire degradation, selective logging) or total removal of the tree cover canopy in each 30 m x 30 m cell for every year from 2001 to 2017^15^. We adopted this definition of forest loss in our study. Therefore, forest loss does not mean exclusively deforestation (complete removal of the forest), but also includes changes such as forest degradation and forest disturbance that may result from different factors^61^.

We used Version 1.5 of the data because it provided the longest time series that is currently available. It is important to note that this version of the forest loss data was created using two different processing algorithms. One was applied for the period from 2001 to 2010 and the second was applied for the period from 2011 to 2017. The second algorithm (which incorporates Landsat 8 data) offers improved change detection, such as better detection of boreal forest loss due to fires and of rotational agricultural leading to clearing of land by smallholders in dry and humid tropical forests. According to recommendations in the user’s guide for the dataset, annual analysis involving the entire time series should be performed with caution. However, Kalamandeen *et al*.^14^ investigated the differences between two Version 1.0 and Version 1.2 of the forest loss data in the same region. Their analysis indicates that during the period where both versions were available, the overall patterns revealed by the two versions were very similar, and they did not apply any further processing to the Hansen *et al*. (2013) dataset. Based on the investigation by Kalamandeen *et al*.^14^ and on the product’s reported accuracy of 99.5% for tropical regions^15^, we assumed that the two periods of forest loss data (before and after 2010) did not differ sufficiently to prevent us from using the entire time series. However, we were aware of the possible bias during our analysis and analyzed the differences between the two periods carefully.

To cover the BLA (5 × 10^6^ km²), we generated two mosaics (forest cover and forest loss) using 12 tiles (each 10° of latitude by 10° of longitude) from Hansen *et al*.’s data. We clipped the two mosaics using the spatial limits of the BLA. We created a forest/no-forest mask for 2000 using a threshold of 30% for tree cover^14,19,46^. Cells that exceeded the 30% forest cover threshold were used as a binary forest/no-forest mask. Using this mask, we extracted only forest loss pixels that were within the forest cover area. To derive the annual datasets, we extracted forest loss data in each year, and for forest cover, we applied the annual forest loss masks to the forest cover dataset as follows:

1 $$\begin{array}{l}F{C}_{2000}-F{L}_{2001}=F{C}_{2001}\\ F{C}_{2001}-F{L}_{2002}=F{C}_{2002}\\ \ldots \\ F{C}_{2016}-F{L}_{2017}=F{C}_{2017}\end{array}$$

where *FC* is the forest cover and *FL* is the forest loss in the indicated years. All data were projected to the World Cylindrical Equal Area projection (EPSG: 54034) to preserve the area information. All these analyses were conducted using ArcGIS 10.6^62^.

### Primary and non-primary forest data

The area of primary forest in 2000 was derived from the PRODES^10^ data. We downloaded PRODES data for the year 2017 from http://www.dpi.inpe.br/prodesdigital/dadosn/mosaicos/2017/. In order to retrieve the primary forest mask, we aggregated all the annual deforestation polygons that were mapped from 2001 to 2017. The resulting PRODES primary forest mask includes disturbed and undisturbed old-growth humid tropical forest within the tropical forest biome^27,28^ for the year 2000. Since we used the PRODES primary forest data as a mask, we adopted the abovementioned description of primary forest as the definition of this forest type in our analysis. This can be justified by the fact that PRODES only identifies annual deforestation of primary forest by clearcutting (it does not account for changes in any other forest type). It is important to note that PRODES’s primary forest mask includes areas that may have been under selective logging or disturbed by understory fires in the past. Furthermore, PRODES treats deforestation as permanent (i.e., the deforested areas are off-limits during the deforestation mapping procedure in the following years) therefore the primary forest mask is in permanent reduction because the newly deforested areas identified in one year (e.g. 2001) are excluded from the mask and thus not analyzed in the following years (e.g. 2002, 2003 and so on). The non-primary forest in our study includes the remaining area of forest cover outside of the PRODES primary forest mask, but within the BLA. In this manner, our definition of non-primary forest includes mainly secondary rainforest areas (forest that is regrowing), savannas (Cerrado), shrublands, dry broadleaf forests, and Pantanal flooded savannas. By applying the PRODES primary forest mask to GFC data, we generated annual GFC forest cover masks for primary forest and non-primary forest. This allowed us to analyze whether the implementation of the anti-deforestation initiatives shifted forest loss processes from primary forest areas (the main targets of the government’s protection initiatives) to non-primary forest areas.

### Forest cover and forest loss within conservation units and indigenous reserves

We used the delimitation of indigenous reserves and conservation units provided by the Environmental Ministry of Brazil to define pixels that belonged to these areas (http://mapas.mma.gov.br/i3geo/datadownload.htm). Using these limits, we were able to analyze forest fragmentation and forest loss separately in conservation units of integral protection areas, which have strict rules for forest conservation, and in conservation units defined as sustainable units, which allow sustainable uses of the forest^32^. Due to overlaps among the conservations units and indigenous reserves, we divided our analysis into four categories: (i) conservation units based on integral protection, (ii) conservation units based on sustainable use, (iii) indigenous reserves and (iv) regions with overlapping categories.

### Forest cover and forest loss metrics

To evaluate the size distribution of the forest cover and forest loss patches, we adopted eight patch size groups: <1 ha; ≥1 and <6.25 ha; ≥6.25 ha and <50 ha; ≥50 ha and <100 ha; ≥100 ha and <200 ha; ≥200 ha and <500 ha; ≥500 ha and <1000 ha; and ≥1000 ha. These size groups were adopted from Kalamandeen *et al*.^14^.

To estimate the forest cover fragmentation, we calculated the number of additional forest fragments in each year by subtracting the number of fragments in the previous year from the number in the new year (e.g., number of fragments in 2001 – number of fragments in 2000 = number of additional fragments in 2001).

To investigate the spatial distribution of forest loss across the BLA, we created a 10 km × 10 km fishnet grid using the Create Fishnet tool provided by the ArcGIS 10.6 software (www.esri.com), and counted forest loss pixels for each cell in the grid (a total of 51220 fishnet grid cells). We defined this grid size based on previous studies^14,49^. We then calculated the percentage of forest loss based on the original forest cover in 2000 for each grid cell as follows:

$$({\rm{Forest}}\,{\rm{loss}}\,{\rm{in}}\,{\rm{yeary}}/{\rm{forest}}\,{\rm{cover}}\,{\rm{in}}\,2000)\times 100 \% $$

We calculated three landscape metrics^63^ for each fishnet grid cell: the number of patches, mean patch size, and edge density (the calculated edge length per unit area). These metrics were calculated for both forest loss and forest cover, both within and outside conservation units and indigenous reserves, and for primary and non-primary forest, for all years from 2001 to 2017.

To calculate the landscape metrics^63^ over a large area and enable cluster processing, we modified a Python script^64^ developed for QGIS^65^. We defined forest loss (*FL*) and forest cover patches as contiguous groups of pixels in the same class (i.e., class 1 for FL pixels in 2001, class 2 for FL pixels in 2002) based on the eight-neighbor rule (i.e., by examining the 8 pixels surrounding each pixel).

### Temporal trend analysis

We applied the non-parametric Mann–Kendall^66^ test to calculate the significance of temporal trends as well their direction (increasing or decreasing) for each landscape metric for each fishnet grid cell:

2 $$S=\mathop{\sum }\limits_{i=0}^{n}\mathop{\sum }\limits_{j=i+1}^{n}{\rm{sign}}\,({y}_{j}-{y}_{i})$$

where *n* is the number of observations and *y* are the observation values in the time series (y_i_ earlier and y_j_ later observations).

Large positive *S* values indicate an increasing trend and large negative *S* values indicate a decreasing trend. Small *S* values (i.e., lay outside the 90% confidence interval) indicated the absence of a trend. To test the null hypothesis that there was no trend, we calculated the *Z*-value using the trend package for the R software (https://cran.r-project.org/web/packages/trend/trend.pdf).

### Neighborhood adjacency

To identify whether the forest loss pixels became spatially closer to each other over time, we counted the cell adjacencies for each pairwise combination of classes and created an adjacency (neighborhood) matrix.

### Hotspot analysis

We estimated spatial clustering of forest loss patches in the BLA using hotspot analysis based on the Getis–Ord *G*_i_* statistic^67^, which identifies aggregation of large and small patches. First, we converted the forest loss patches in each year into points, and for each point, we assigned the area of the patch as that point’s attribute value. We used the ArcGIS^62^ hotspot analysis tool on the point data to calculate the *G*_i_* statistic using a fixed 10-km distance. This distance defined the neighborhood (<10 km) and non-neighborhood (≥10 km) points. The patch areas were used as the input value for the *G*_i_* statistic. The *G*_i_* statistic sums the area values of each point and its neighboring points (<10 km) and compared this sum with the overall sum of all features in the study area. If the sum of the point and its neighbors is very different from the overall sum, a *Z*-score and the associated *P* values are calculated. Forest loss patches, with either large or small area, can formed a significant spatial cluster. Points with a high positive *Z*-score (≥1.645) and a significant *P* value (i.e., hot spots) represent clusters of large forest loss areas, whereas points with a low negative *Z*-score (≤–1.645) and a significant *P* value (i.e., cold spots) represent clusters of small forest loss areas. We performed the hotspot analysis separately for each year.

### Active fire data

To estimate the forest loss potentially caused by fire, we used the Fire Information for Resource Management System (FIRMS) active fire product^68^ which is derived from the MODIS sensor aboard the NASA Aqua and Terra satellites. We used the MCD14ML (collection 6) product for active fire data from 2001 to 2017. This data is produced based on the daily MODIS middle-infrared and thermal infrared bands, which are compared with reference data in order to identify pixels with an active fire. We used the definition “potentially caused by fire” for two reasons. The first one relates to the difference in spatial resolution between the forest loss data (30 m) and the MODIS-based product (1 km), which is a limitation of our results because a fire could be located in any area within the 1-km MODIS pixel. The second relates to the fact that fire is used as a management tool for purposes such as burning the residual vegetation that remains or begins to grow after deforestation, which means that it was not directly responsible for the forest loss. We combined the annual (FIRMS) active fire points with the forest loss patches (polygons) during the same year in order to identify forest loss patches that contained one or more FIRMS active fire points within their borders. By summarizing the area of these forest loss patches, we obtained the total area of forest loss that had been potentially caused by fire.

## Supplementary information

Supplementary Information.

## Data Availability

The forest products used as input in our study can be found in the respective references. The forest cover, forest loss and active fire data resulting from our analysis are available upon request from the corresponding author. Supplementary scripts developed and used in the analysis can be found in the following repository https://github.com/LandscapeGeoinformatics/LandscapeMetrics_and_TrendCalc.
