# Supplementary material for: Increasing fragmentation of forest cover in Brazil’s Legal Amazon from 2001 to 2017
Source: Sci Rep. 2020 Apr 2;10:5803. doi: 10.1038/s41598-020-62591-x (PMC7118152; doi:10.1038/s41598-020-62591-x)
Supplement: Supplementary file 1 — Supplementary Information. [file 41598_2020_62591_MOESM1_ESM.docx]

**Increasing fragmentation of forest cover in Brazil’s Legal Amazon from 2001 to 2017**

**Bruno Montibeller*^1^, Alexander Kmoch^1^, Holger Virro^1^, Ülo Mander^1^, Evelyn Uuemaa^1^**

**SUPPLEMENTARY MATERIAL**

**
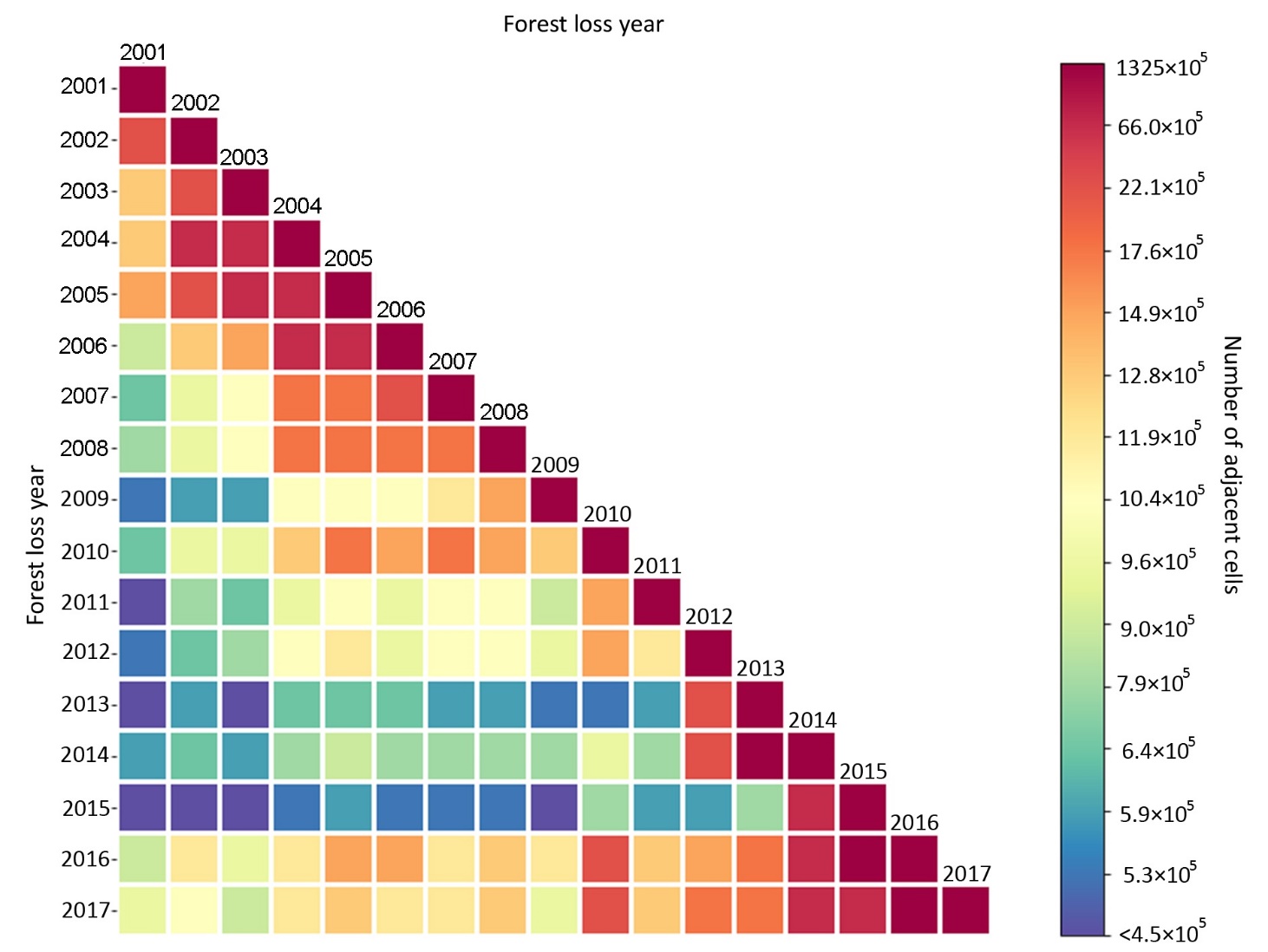
Figure S1. Adjacency matrix for annual forest loss cells.** Spatial and temporal adjacency matrix for cells in which forest loss occurred in the Brazilian Legal Amazon. Each cell shows the number of adjacencies (counted using the 8-neighbor rule) with other cells in which forest loss occurred in respective years.

**
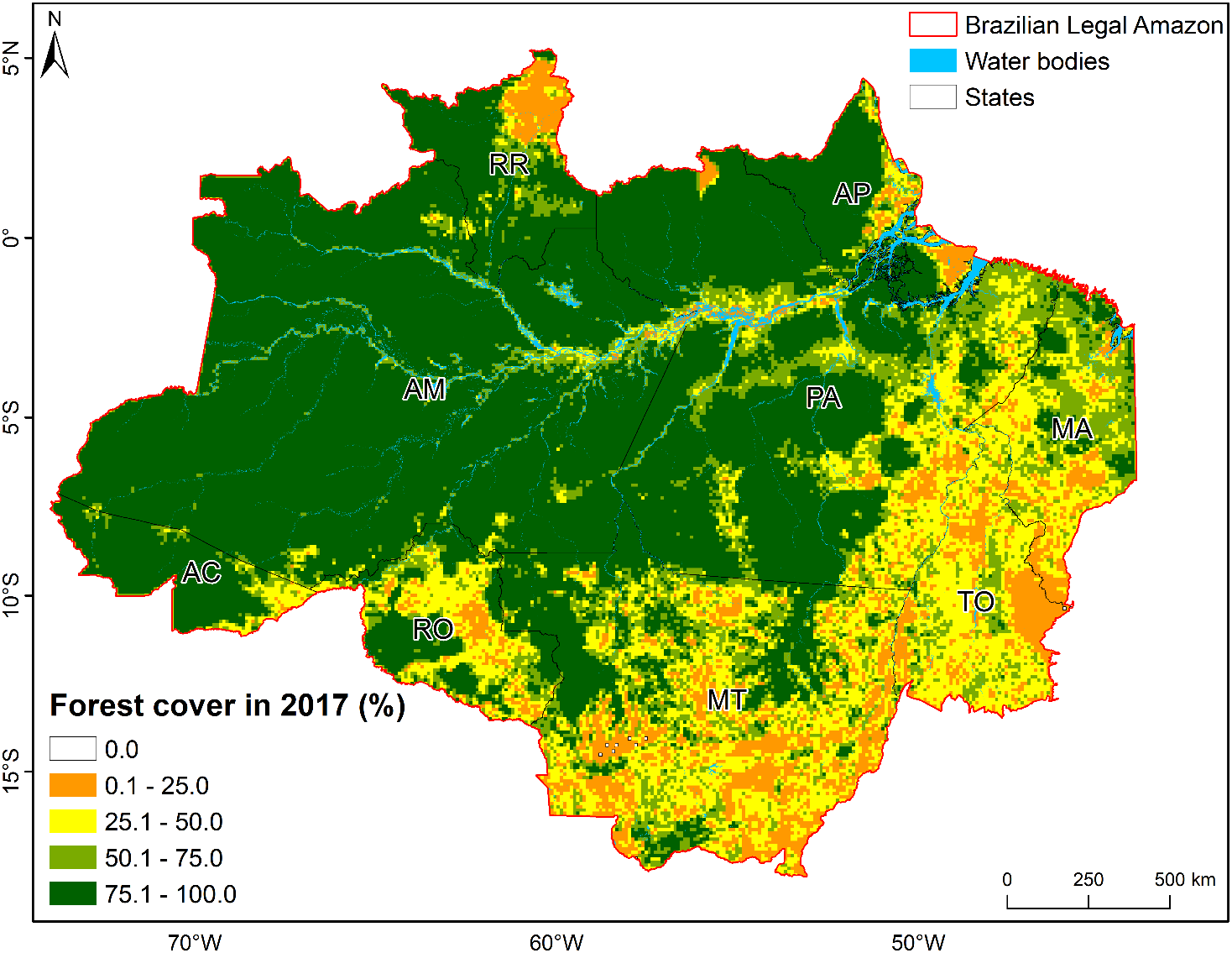
**

**Figure S2. Forest dynamics in the Brazilian Legal Amazon (BLA).** Percentage forest cover (FC) in each 10 km × 10 km grid cell in 2017.

**Table S1***.* Changes in the forest loss area, number of patches, mean patch area, and edge density within and outside of conservation units (CU) and indigenous reserves (IR) from 2001 to 2017 in the Brazilian Legal Amazon (BLA). The statistical significance of the trend was estimated using the Mann–Kendall test.

| **Year** | **Forest loss area (ha)** | | | **Number of patches with forest loss** | | | **Mean patch size (ha)** | | | **Edge density (m/ha)** | | |
| --- | --- | --- | --- | --- | --- | --- | --- | --- | --- | --- | --- | --- |
|  | CU/IR | Outside CU/IR | Total BLA | CU/IR | Outside CU/IR | Total BLA | CU/IR | Outside CU/IR | Total BLA | CU/IR | Outside CU/IR | Total BLA |
| 2001 | 121927 | 1827145 | 1949072 | 125091 | 1026664 | 1151755 | 0.97 | 1.78 | 1.69 | 370 | 296 | 300 |
| 2002 | 144824 | 2530175 | 2674999 | 131735 | 1031684 | 1163419 | 1.09 | 2.45 | 2.29 | 350 | 243 | 248 |
| 2003 | 131989 | 2309688 | 2441677 | 111144 | 827969 | 939113 | 1.10 | 2.79 | 2.6 | 339 | 224 | 230 |
| 2004 | 223586 | 2795943 | 3019529 | 131631 | 1047388 | 1179019 | 1.70 | 2.67 | 2.56 | 269 | 232 | 234 |
| 2005 | 194239 | 2530826 | 2725065 | 145461 | 1134420 | 1279881 | 1.33 | 2.23 | 2.12 | 321 | 266 | 269 |
| 2006 | 169775 | 1916975 | 2086751 | 142392 | 945862 | 1088254 | 1.19 | 2.03 | 1.91 | 347 | 281 | 286 |
| 2007 | 175686 | 1616784 | 1792471 | 158342 | 944204 | 1102546 | 1.11 | 1.71 | 1.62 | 362 | 308 | 312 |
| 2008 | 182654 | 1471661 | 1654315 | 159036 | 910073 | 1069109 | 1.15 | 1.62 | 1.54 | 362 | 322 | 326 |
| 2009 | 114222 | 1045362 | 1159584 | 140389 | 774336 | 914725 | 0.81 | 1.35 | 1.26 | 441 | 356 | 364 |
| 2010 | 206384 | 1590749 | 1797133 | 221064 | 1173501 | 1394565 | 0.93 | 1.36 | 1.28 | 422 | 359 | 365 |
| 2011 | 148898 | 1116499 | 1265397 | 180804 | 852507 | 1033311 | 0.82 | 1.31 | 1.22 | 450 | 365 | 374 |
| 2012 | 212082 | 1587211 | 1799293 | 327775 | 1231042 | 1558817 | 0.64 | 1.29 | 1.15 | 469 | 349 | 363 |
| 2013 | 139610 | 1115713 | 1255323 | 209713 | 1212425 | 1422138 | 0.66 | 0.92 | 0.88 | 485 | 428 | 434 |
| 2014 | 95721 | 1680500 | 1776221 | 317908 | 1751056 | 2068964 | 0.61 | 0.96 | 0.85 | 1081 | 415 | 450 |
| 2015 | 185065 | 1374765 | 1559830 | 168618 | 970968 | 1139586 | 1.10 | 1.42 | 1.36 | 404 | 372 | 375 |
| 2016 | 740297 | 3450788 | 4191085 | 504904 | 2362359 | 2867263 | 1.47 | 1.46 | 1.46 | 349 | 352 | 351 |
| 2017 | 592976 | 2878679 | 3471655 | 516283 | 2463468 | 2979751 | 1.15 | 1.17 | 1.16 | 397 | 391 | 392 |
| sig. level | ns | ns | ns | *** | * | * | ns | *** | *** | * | *** | ns |
| *z*-value | 1.36 | -0.95 | -0.95 | 4.16 | 2.10 | 2.18 | -0.95 | -3.58 | -3.58 | 2.43 | 3.83 | 0.70 |

Significance: ns, not statistically significant; * <0.05; **<0.01; ***<0.001

**Table S2***.* Changes in the forest loss area, number of patches with forest loss, mean patch size, and edge density within integral protection (IP) and sustainable use (SU) conservation units, within indigenous reserves (IR), and within areas that overlap between categories (Overl.) from 2001 to 2017 in the Brazilian Legal Amazon (BLA). The statistical significance of the trend was estimated using the Mann–Kendall test.

| **Year** | **Forest loss area (ha)** | | | | **Number of patches with forest loss** | | | | **Mean patch size (ha)** | | | | **Edge density (m/ha)** | | | |
| --- | --- | --- | --- | --- | --- | --- | --- | --- | --- | --- | --- | --- | --- | --- | --- | --- |
|  | IP | SU | IR | Overl. | IP | SU | IR | Overl. | IP | SU | IR | Overl. | IP | SU | IR | Overl. |
| 2001 | 12842.82 | 76159.62 | 31997.52 | 770.67 | 14158 | 62235 | 46606 | 2210 | 0.90 | 1.22 | 0.68 | 0.34 | 359.03 | 324.45 | 477.55 | 699.91 |
| 2002 | 21179.70 | 80306.73 | 42585.93 | 756.45 | 13777 | 71113 | 45107 | 1845 | 1.53 | 1.13 | 0.94 | 0.41 | 281.16 | 351.38 | 377.94 | 648.26 |
| 2003 | 17987.04 | 72103.32 | 39928.86 | 1830.96 | 10981 | 57863 | 38409 | 3976 | 1.63 | 1.24 | 1.03 | 0.46 | 284.60 | 336.50 | 356.81 | 590.02 |
| 2004 | 32247.90 | 128251.44 | 61481.61 | 1532.52 | 16480 | 65247 | 47505 | 2426 | 1.95 | 1.96 | 1.29 | 0.63 | 245.79 | 252.30 | 313.46 | 436.46 |
| 2005 | 23605.65 | 126599.94 | 43154.73 | 841.32 | 17010 | 80405 | 46547 | 1568 | 1.38 | 1.57 | 0.92 | 0.53 | 316.69 | 295.78 | 395.18 | 515.69 |
| 2006 | 17892.18 | 104987.88 | 45406.62 | 1213.02 | 14875 | 77639 | 47164 | 2759 | 1.20 | 1.35 | 0.96 | 0.43 | 344.10 | 321.61 | 401.82 | 599.89 |
| 2007 | 15327.36 | 102144.96 | 56191.77 | 1700.91 | 13094 | 75955 | 65703 | 3616 | 1.17 | 1.34 | 0.85 | 0.47 | 342.98 | 311.18 | 452.24 | 606.77 |
| 2008 | 12024.81 | 117259.38 | 52343.82 | 926.01 | 14052 | 82377 | 60175 | 2435 | 0.85 | 1.42 | 0.86 | 0.38 | 404.06 | 325.94 | 427.91 | 660.64 |
| 2009 | 7457.67 | 75476.88 | 29351.52 | 1882.89 | 12820 | 81159 | 43190 | 3290 | 0.58 | 0.92 | 0.67 | 0.57 | 506.92 | 409.73 | 499.77 | 542.77 |
| 2010 | 17624.52 | 86567.94 | 98057.61 | 3505.86 | 21488 | 89509 | 102693 | 7451 | 0.82 | 0.96 | 0.95 | 0.47 | 423.07 | 406.39 | 429.84 | 639.83 |
| 2011 | 11186.10 | 74752.47 | 59171.04 | 3087.81 | 18585 | 79664 | 77340 | 5363 | 0.6 | 0.93 | 0.76 | 0.57 | 501.89 | 418.50 | 478.09 | 561.80 |
| 2012 | 17265.69 | 101311.02 | 89002.08 | 4215.33 | 48960 | 139043 | 130693 | 9273 | 0.35 | 0.72 | 0.68 | 0.45 | 643.07 | 443.51 | 1.47 | 579.67 |
| 2013 | 7777.53 | 88078.14 | 40758.39 | 2807.55 | 18000 | 117165 | 69058 | 5478 | 0.43 | 0.75 | 0.59 | 0.51 | 611.71 | 446.89 | 538.09 | 575.69 |
| 2014 | 10083.60 | 131093.73 | 52048.62 | 2313.36 | 20016 | 190527 | 101716 | 5643 | 0.5 | 0.69 | 0.51 | 0.40 | 585.48 | 489.74 | 608.05 | 697.71 |
| 2015 | 10415.43 | 123186.51 | 49954.23 | 1437.84 | 9705 | 95171 | 61630 | 2075 | 0.07 | 1.29 | 0.81 | 0.69 | 376.62 | 356.74 | 520.60 | 549.49 |
| 2016 | 71095.41 | 317991.06 | 327098.43 | 21939.12 | 31943 | 248134 | 209775 | 15121 | 2.22 | 1.28 | 1.55 | 1.45 | 274.25 | 361.10 | 352.65 | 375.06 |
| 2017 | 43841.52 | 243335.43 | 289419.03 | 15903.99 | 34257 | 256238 | 213614 | 12194 | 1.28 | 0.94 | 1.35 | 1.3 | 365.00 | 430.22 | 373.01 | 428.40 |
| sig. level | ns | ns | * | ** | * | *** | *** | ** | * | ns | ns | * | * | ** | ns | ns |
| z-value | -0.95 | 1.77 | 2.27 | 3.17 | 2.02 | 4.33 | 3.42 | 3.01 | -2.27 | -1.85 | -0.74 | 2.23 | 2.02 | 2.92 | 1.19 | -1.52 |

Significance: ns, not statistically significant; * <0.05; **<0.01; ***<0.001

**Table S3***.* Changes in the number of forest loss patches in each size category from 2001 to 2017 outside and within conservation units (CU) and indigenous reserves (IR) in the Brazilian Legal Amazon (BLA). The statistical significance of the trend was estimated using the Mann–Kendall test.

|  | **Number of forest loss patches** | | | | | | | | | | | | | | | |
| --- | --- | --- | --- | --- | --- | --- | --- | --- | --- | --- | --- | --- | --- | --- | --- | --- |
| **Year** | **<1 ha** | | **≥1 and <6.25 ha** | | **≥6.25 and <50 ha** | | **≥50 and <100 ha** | | **≥100 and <200 ha** | | **≥200 and <500 ha** | | **≥500 and <1000 ha** | | **≥1000 ha** | |
|  | Outside CU/IR | CU/IR | Outside CU/IR | CU/IR | Outside CU/IR | CU/IR | Outside CU/IR | CU/IR | Outside CU/IR | CU/IR | Outside CU/IR | CU/IR | Outside CU/IR | CU/IR | Outside CU/IR | CU/IR |
| 2001 | 822810 | 110219 | 160226 | 12698 | 39275 | 1912 | 2526 | 141 | 1067 | 73 | 594 | 37 | 128 | 9 | 38 | 2 |
| 2002 | 798278 | 114353 | 176552 | 14815 | 50001 | 2268 | 3715 | 175 | 1831 | 69 | 949 | 43 | 263 | 5 | 95 | 7 |
| 2003 | 623987 | 95471 | 152733 | 12947 | 45181 | 2409 | 3340 | 183 | 1502 | 87 | 856 | 42 | 255 | 2 | 115 | 3 |
| 2004 | 793251 | 111223 | 190588 | 16363 | 56020 | 3464 | 4125 | 300 | 1995 | 144 | 1025 | 99 | 271 | 25 | 113 | 13 |
| 2005 | 860853 | 121914 | 205356 | 18990 | 62102 | 4111 | 3807 | 283 | 1419 | 107 | 673 | 48 | 160 | 4 | 50 | 4 |
| 2006 | 709243 | 119269 | 182994 | 19163 | 49388 | 3576 | 2795 | 255 | 969 | 91 | 355 | 36 | 89 | 1 | 29 | 1 |
| 2007 | 728839 | 134841 | 170730 | 19742 | 41477 | 3342 | 2027 | 267 | 755 | 103 | 273 | 38 | 65 | 8 | 18 | 1 |
| 2008 | 696205 | 133532 | 172356 | 21255 | 38970 | 3909 | 1699 | 219 | 564 | 81 | 219 | 33 | 45 | 4 | 15 | 3 |
| 2009 | 598593 | 119359 | 146333 | 18630 | 28014 | 2286 | 968 | 68 | 294 | 31 | 111 | 14 | 18 | 1 | 5 | 0 |
| 2010 | 943555 | 190889 | 187582 | 25721 | 39692 | 4112 | 1760 | 232 | 624 | 68 | 225 | 32 | 43 | 6 | 20 | 4 |
| 2011 | 681772 | 156946 | 140121 | 20516 | 28924 | 3156 | 1116 | 125 | 397 | 39 | 144 | 19 | 23 | 3 | 10 | 0 |
| 2012 | 1024695 | 298845 | 165107 | 24849 | 38224 | 3817 | 1939 | 141 | 667 | 76 | 289 | 33 | 64 | 9 | 27 | 5 |
| 2013 | 1032420 | 184189 | 153581 | 23258 | 24994 | 2125 | 935 | 82 | 320 | 34 | 132 | 18 | 31 | 4 | 12 | 3 |
| 2014 | 1537560 | 287516 | 177872 | 27349 | 33106 | 2784 | 584 | 1157 | 578 | 58 | 283 | 41 | 47 | 2 | 26 | 1 |
| 2015 | 778175 | 141971 | 159314 | 23577 | 30922 | 2773 | 1578 | 177 | 630 | 58 | 292 | 46 | 49 | 11 | 8 | 5 |
| 2016 | 1998240 | 429389 | 297900 | 64001 | 59953 | 10172 | 3524 | 695 | 1529 | 360 | 802 | 183 | 240 | 61 | 171 | 43 |
| 2017 | 2103273 | 442321 | 301088 | 63560 | 54192 | 9378 | 2824 | 581 | 1244 | 256 | 585 | 117 | 176 | 38 | 86 | 32 |
| Sig. level | * | *** | ns | *** | ns | ns | * | ns | ns | ns | ns | ns | ns | ns | ns | ns |
| *z*-value | 2.02 | 4.16 | 0.45 | 4.41 | -1.36 | 1.52 | -2.02 | 0.58 | -1.61 | -0.49 | -1.36 | 0.00 | -1.52 | 0.95 | -1.36 | 0.75 |

Significance: ns, not statistically significant; * <0.05; **<0.01; ***<0.001

**Table S4***.* Changes in the forest loss area, number of patches, mean patch size, and edge density in primary forest (PF) and non-primary forest (NPF) from 2001 to 2017 in the Brazilian Legal Amazon (BLA). The statistical significance of the trend was estimated by using the Mann-Kendall test.

| **year** | **Forest loss area (ha)** | | **Number of patches** | | **Mean patch size (ha)** | | **Edge density (m/ha)** | |
| --- | --- | --- | --- | --- | --- | --- | --- | --- |
|  | PF | NPF | PF | NPF | PF | NPF | PF | NPF |
| 2001 | 1044024 | 905781.41 | 560370 | 1137372 | 1.86 | 0.79 | 239.26 | 423.04 |
| 2002 | 1629600 | 1046159.8 | 640278 | 1094269 | 2.55 | 0.95 | 201.16 | 379.44 |
| 2003 | 1556324 | 886223.16 | 554282 | 828964 | 2.81 | 1.07 | 191.80 | 352.14 |
| 2004 | 2027451 | 993038.04 | 710907 | 1016412 | 2.85 | 0.98 | 194.63 | 372.69 |
| 2005 | 1860648 | 865212.92 | 816891 | 1073266 | 2.28 | 0.81 | 229.84 | 425.20 |
| 2006 | 1424190 | 663069.96 | 705875 | 850536 | 2.01 | 0.78 | 248.14 | 432.42 |
| 2007 | 1175793 | 617166.45 | 699016 | 836076 | 1.68 | 0.74 | 271.97 | 439.24 |
| 2008 | 1092383 | 562504.14 | 709859 | 779915 | 1.54 | 0.72 | 294.36 | 442.80 |
| 2009 | 705741.1 | 454045.5 | 572221 | 677032 | 1.24 | 0.67 | 332.28 | 465.28 |
| 2010 | 1165887 | 632261.34 | 910018 | 1029191 | 1.28 | 0.61 | 326.12 | 484.18 |
| 2011 | 802684.1 | 463262.4 | 696864 | 718901 | 1.15 | 0.65 | 346.05 | 467.34 |
| 2012 | 1126553 | 673819.29 | 974476 | 1118980 | 1.15 | 0.6 | 322.38 | 468.87 |
| 2013 | 690709 | 565051.59 | 888176 | 1018123 | 0.78 | 0.55 | 407.72 | 497.95 |
| 2014 | 1031833 | 745332.12 | 1462410 | 1416373 | 0.7 | 0.53 | 424.43 | 513.66 |
| 2015 | 903117.9 | 657571.32 | 782325 | 815454 | 1.15 | 0.8 | 351.09 | 438.97 |
| 2016 | 2944328 | 1264807.7 | 2358141 | 1726480 | 1.25 | 0.73 | 327.66 | 445.70 |
| 2017 | 2275951 | 1204771.6 | 2317551 | 1823085 | 0.98 | 0.66 | 366.40 | 473.27 |
| sig. level | ns | ns | ** | ns | *** | ** | *** | *** |
| *z*-value | -1.03 | -0.62 | 3.17 | 0.62 | -3.88 | -3.17 | 4.00 | 3.58 |

Significance: ns, not significant; * <0.05; **<0.01; ***<0.001

**Table S5.** Changes in the number of forest loss patches in primary forest (PF) and non-primary forest (NPF) in each size category from 2000 to 2017 within the Brazilian Legal Amazon (BLA). The statistical significance of the trend was estimated by using the Mann–Kendall test.

| **Year** | **Number of forest loss patches** | | | | | | | | | | | | | | | |
| --- | --- | --- | --- | --- | --- | --- | --- | --- | --- | --- | --- | --- | --- | --- | --- | --- |
|  | **<1 ha** | | **≥1 and <6.25 ha** | | **≥6.25 and <50 ha** | | **≥50 and <100 ha** | | **≥100 and <200 ha** | | **≥200 and <500 ha** | | **≥500 and <1000 ha** | | **≥1000 ha** | |
|  | PF | NPF | PF | NPF | PF | NPF | PF | NPF | PF | NPF | PF | NPF | PF | NPF | PF | NPF |
| 2001 | 461765 | 993155 | 75615 | 123479 | 20088 | 19688 | 1623 | 659 | 734 | 244 | 421 | 123 | 95 | 18 | 29 | 6 |
| 2002 | 513195 | 936257 | 93110 | 133038 | 29194 | 23473 | 2482 | 938 | 1311 | 379 | 725 | 134 | 184 | 37 | 77 | 13 |
| 2003 | 433489 | 703149 | 87821 | 105535 | 28632 | 18757 | 2345 | 918 | 1084 | 364 | 637 | 183 | 192 | 44 | 82 | 14 |
| 2004 | 552376 | 867702 | 114775 | 125743 | 37954 | 21412 | 3079 | 926 | 1567 | 401 | 821 | 186 | 239 | 33 | 96 | 9 |
| 2005 | 634324 | 921449 | 133740 | 130764 | 44024 | 20208 | 2953 | 595 | 1092 | 178 | 568 | 65 | 144 | 7 | 46 | 0 |
| 2006 | 546120 | 728427 | 120366 | 106988 | 35942 | 14530 | 2253 | 419 | 793 | 122 | 307 | 41 | 67 | 9 | 27 | 0 |
| 2007 | 555063 | 728488 | 111655 | 94402 | 29761 | 12532 | 1649 | 422 | 586 | 166 | 233 | 56 | 56 | 9 | 13 | 1 |
| 2008 | 558177 | 680441 | 120192 | 88426 | 29525 | 10505 | 1319 | 339 | 450 | 132 | 152 | 55 | 33 | 13 | 11 | 4 |
| 2009 | 455335 | 591685 | 96636 | 76783 | 19338 | 8195 | 647 | 235 | 170 | 99 | 80 | 28 | 14 | 5 | 1 | 2 |
| 2010 | 749701 | 919132 | 129024 | 97852 | 29221 | 11638 | 1372 | 381 | 476 | 139 | 171 | 41 | 35 | 5 | 18 | 3 |
| 2011 | 574111 | 640918 | 100409 | 68975 | 21207 | 8520 | 759 | 327 | 262 | 105 | 95 | 44 | 15 | 11 | 6 | 1 |
| 2012 | 832841 | 1016882 | 112334 | 88816 | 27247 | 12456 | 1289 | 544 | 472 | 194 | 222 | 72 | 47 | 12 | 24 | 4 |
| 2013 | 776024 | 925912 | 96481 | 81977 | 14890 | 9641 | 494 | 388 | 167 | 142 | 89 | 50 | 21 | 9 | 10 | 4 |
| 2014 | 1326206 | 1312519 | 114047 | 90522 | 20646 | 12407 | 946 | 584 | 345 | 226 | 169 | 102 | 32 | 10 | 19 | 3 |
| 2015 | 660842 | 713277 | 100948 | 88722 | 18889 | 12569 | 981 | 566 | 409 | 218 | 206 | 92 | 42 | 9 | 8 | 1 |
| 2016 | 2069356 | 1536265 | 236252 | 165029 | 46959 | 23616 | 3003 | 986 | 1377 | 376 | 800 | 165 | 228 | 33 | 166 | 10 |
| 2017 | 2055462 | 1634163 | 218264 | 166202 | 39798 | 21345 | 2263 | 851 | 1008 | 324 | 504 | 156 | 165 | 34 | 87 | 10 |
| Sig. level | *** | ns | ns | ns | ns | ns | ns | ns | ns | ns | ns | ns | ns | ns | ns | ns |
| *z*-value | 3.83 | 1.03 | 1.69 | -1.03 | -0.62 | -1.03 | -1.69 | -0.54 | -1.77 | -0.12 | -1.52 | 0.00 | -1.36 | -0.46 | -1.03 | 0.00 |

Significance: ns, not significant; * <0.05; **<0.01; ***<0.001

**Table S6***.* Changes in the forest cover area, number of patches, mean patch area, and edge density within and outside of conservation units (CU) and indigenous reserves (IR) in the Brazilian Legal Amazon (BLA).

| **Year** | **Forest cover area (ha)** | | | **Number of patches** | | | **Mean patch size (ha)** | | | **Edge density (m/ha)** | | |
| --- | --- | --- | --- | --- | --- | --- | --- | --- | --- | --- | --- | --- |
|  | CU/IR | Outside CU/IR | Total BLA | CU/IR | Outside CU/IR | Total BLA | CU/IR | Outside CU/IR | Total BLA | CU/IR | Outside CU/IR | Total BLA |
| 2000 | 208909053 | 198207199 | 407116252 | 886192 | 4435939 | 5249458 | 235.73 | 44.68 | 77.56 | 7.87 | 37.62 | 21.70 |
| 2001 | 208781715 | 196385708 | 405167422 | 889817 | 4493764 | 5310375 | 234.63 | 43.7 | 76.3 | 8.01 | 38.68 | 22.23 |
| 2002 | 208628116 | 193864665 | 402492781 | 897834 | 4630336 | 5454169 | 232.36 | 41.87 | 73.8 | 8.18 | 40.29 | 22.99 |
| 2003 | 208484311 | 191566905 | 400051216 | 905893 | 4748639 | 5579866 | 230.14 | 40.34 | 71.7 | 8.33 | 41.73 | 23.67 |
| 2004 | 208245326 | 188786612 | 397031938 | 920624 | 4901124 | 5746236 | 226.2 | 38.52 | 69.1 | 8.54 | 43.53 | 24.52 |
| 2005 | 208040178 | 186266790 | 394306968 | 935806 | 5104812 | 5964173 | 222.31 | 36.49 | 66.12 | 8.73 | 45.28 | 25.34 |
| 2006 | 207861003 | 184359398 | 392220401 | 949177 | 5272192 | 6144108 | 219 | 34.97 | 63.84 | 8.91 | 46.77 | 26.05 |
| 2007 | 207679370 | 182748762 | 390428132 | 963435 | 5419501 | 6304938 | 215.56 | 33.72 | 61.93 | 9.13 | 48.16 | 26.74 |
| 2008 | 207490411 | 181283460 | 388773871 | 979798 | 5556917 | 6458000 | 211.76 | 32.62 | 60.2 | 9.32 | 49.34 | 27.32 |
| 2009 | 207372401 | 180241930 | 387614331 | 989634 | 5662435 | 6572766 | 209.54 | 31.83 | 58.98 | 9.47 | 50.31 | 27.80 |
| 2010 | 207157696 | 178659541 | 385817237 | 1012055 | 5823223 | 6755043 | 204.69 | 30.68 | 57.12 | 9.75 | 51.66 | 28.49 |
| 2011 | 207003015 | 177549076 | 384552091 | 1030344 | 5951066 | 6900480 | 200.9 | 29.83 | 55.73 | 9.95 | 52.59 | 28.97 |
| 2012 | 206782717 | 175970644 | 382753360 | 1057484 | 6162589 | 7138184 | 195.54 | 28.55 | 53.62 | 10.28 | 54.15 | 29.79 |
| 2013 | 206638472 | 174859702 | 381498174 | 1072717 | 6340725 | 7330812 | 192.63 | 27.57 | 52.04 | 10.52 | 55.81 | 30.61 |
| 2014 | 206432132 | 173290090 | 379722222 | 1097914 | 6607700 | 7621342 | 188.02 | 26.23 | 49.83 | 10.83 | 57.70 | 31.55 |
| 2015 | 206240615 | 171921850 | 378162465 | 1121444 | 6852378 | 7888490 | 183.9 | 25.09 | 47.94 | 11.05 | 59.21 | 32.28 |
| 2016 | 205396783 | 168574630 | 373971413 | 1214130 | 7314753 | 8440765 | 169.17 | 23.05 | 44.31 | 11.96 | 62.98 | 34.29 |
| 2017 | 204734709 | 165765122 | 370499830 | 1274832 | 7665207 | 8849746 | 160.6 | 21.63 | 41.87 | 12.54 | 65.41 | 35.53 |
| sig. Level | *** | *** | *** | *** | *** | *** | *** | *** | *** | *** | *** | *** |
| *z*-value | -5.76 | -5.76 | -5.76 | -5.76 | -5.76 | -5.76 | -5.76 | -5.76 | -5.76 | 5.76 | 5.76 | 5.76 |

Significance: ***<0.001

**Table S7**. Changes in the number of forest cover fragments outside and within conservation units (CU) and indigenous reserves (IR) in each size category from 2000 to 2017 within the Brazilian Legal Amazon (BLA). The significance of the trend was estimated using the Mann–Kendall test.

|  | **Number of fragments** | | | | | | | | | | | | | | | |
| --- | --- | --- | --- | --- | --- | --- | --- | --- | --- | --- | --- | --- | --- | --- | --- | --- |
| **Year** | **<1 ha** | | **≥1 and <6.25 ha** | | **≥6.25 and <50 ha** | | **≥50 and <100 ha** | | **≥100 and <200 ha** | | **≥200 and <500 ha** | | **≥500 and <1000 ha** | | **≥1000 ha** | |
|  | CU/IR | Outside CU/IR | CU/IR | Outside CU/IR | CU/IR | Outside CU/IR | CU/IR | Outside CU/IR | CU/IR | Outside CU/IR | CU/IR | Outside CU/IR | CU/IR | Outside CU/IR | CU/IR | Outside CU/IR |
| 2000 | 759579 | 3738548 | 95396 | 530017 | 25309 | 137103 | 2458 | 13243 | 1327 | 7483 | 958 | 5160 | 353 | 2028 | 812 | 2357 |
| 2001 | 762594 | 3783176 | 95869 | 539364 | 25433 | 140272 | 2466 | 13504 | 1328 | 7636 | 964 | 5349 | 355 | 2056 | 808 | 2407 |
| 2002 | 769576 | 3896819 | 96705 | 556063 | 25587 | 145446 | 2480 | 13928 | 1340 | 7925 | 974 | 5550 | 363 | 2114 | 809 | 2491 |
| 2003 | 776661 | 3995589 | 97496 | 570353 | 25747 | 149878 | 2487 | 14376 | 1352 | 8080 | 977 | 5683 | 360 | 2152 | 813 | 2528 |
| 2004 | 789651 | 4124776 | 98878 | 587085 | 26083 | 155323 | 2495 | 14861 | 1360 | 8338 | 980 | 5894 | 363 | 2227 | 814 | 2620 |
| 2005 | 803190 | 4299718 | 100202 | 608306 | 26336 | 161416 | 2512 | 15515 | 1380 | 8712 | 992 | 6134 | 371 | 2299 | 823 | 2712 |
| 2006 | 815253 | 4445648 | 101248 | 624082 | 26561 | 165961 | 2528 | 15995 | 1383 | 9014 | 998 | 6335 | 381 | 2362 | 825 | 2795 |
| 2007 | 828140 | 4575155 | 102298 | 637363 | 26843 | 169653 | 2556 | 16344 | 1391 | 9206 | 1006 | 6519 | 376 | 2420 | 825 | 2841 |
| 2008 | 842889 | 4697591 | 103568 | 648488 | 27130 | 172742 | 2566 | 16583 | 1411 | 9482 | 1022 | 6650 | 388 | 2470 | 824 | 2911 |
| 2009 | 851929 | 4793897 | 104236 | 655570 | 27223 | 174446 | 2592 | 16720 | 1418 | 9630 | 1015 | 6738 | 398 | 2490 | 823 | 2944 |
| 2010 | 872768 | 4942548 | 105564 | 664547 | 27444 | 176725 | 2604 | 17113 | 1432 | 9852 | 1020 | 6874 | 399 | 2564 | 824 | 3000 |
| 2011 | 889790 | 5062215 | 106624 | 670546 | 27611 | 178302 | 2629 | 17372 | 1437 | 9993 | 1025 | 6964 | 414 | 2592 | 814 | 3082 |
| 2012 | 914929 | 5259195 | 108293 | 682060 | 27900 | 180514 | 2663 | 17691 | 1441 | 10232 | 1034 | 7062 | 412 | 2673 | 812 | 3162 |
| 2013 | 929059 | 5421286 | 109264 | 695115 | 28009 | 183008 | 2675 | 17902 | 1445 | 10345 | 1034 | 7152 | 413 | 2717 | 818 | 3200 |
| 2014 | 952350 | 5663416 | 110859 | 715153 | 28268 | 186966 | 2702 | 18302 | 1463 | 10546 | 1032 | 7330 | 423 | 2750 | 817 | 3237 |
| 2015 | 973900 | 5883701 | 112509 | 734133 | 28551 | 191540 | 2724 | 18737 | 1462 | 10746 | 1052 | 7471 | 428 | 2765 | 818 | 3285 |
| 2016 | 1055668 | 6285492 | 121502 | 781392 | 30256 | 202874 | 2837 | 19656 | 1521 | 11240 | 1095 | 7792 | 429 | 2888 | 822 | 3419 |
| 2017 | 1108012 | 6584111 | 128254 | 820470 | 31653 | 213345 | 2933 | 20753 | 1575 | 11758 | 1137 | 8188 | 439 | 2997 | 829 | 3585 |
| Sig. level | *** | *** | *** | *** | *** | *** | *** | *** | *** | *** | *** | *** | *** | *** | ns | *** |
| *z*-value | 5.56 | 5.56 | 5.56 | 5.56 | 5.56 | 5.56 | 5.56 | 5.56 | 5.48 | 5.56 | 5.19 | 5.56 | 5.19 | 5.56 | 1.28 | 5.56 |

Significance: ns, not significant; *** < 0.001

**Table S8***.* Changes in the forest area, number of patches, mean patch size, and edge density within integral protection (IP) and sustainable use (SU) conservation units, within indigenous reserves (IR), and within areas that overlap categories (Overl.) from 2001 to 2017 in the Brazilian Legal Amazon (BLA).

| **Year** | **Forest area (ha)** | | | | **Number of patches** | | | | **Mean patch size (ha)** | | | | **Edge density (m/ha)** | | | |
| --- | --- | --- | --- | --- | --- | --- | --- | --- | --- | --- | --- | --- | --- | --- | --- | --- |
|  | IP | SU | IR | Overl. | IP | SU | IR | Overl. | IP | SU | IR | Overl. | IP | SU | IR | Overl. |
| 2000 | 36049280.85 | 66825712.53 | 96301268.91 | 10160283.7 | 175742 | 387801 | 313859 | 29575 | 205.12 | 172.31 | 306.83 | 343.54 | 8.16 | 11.35 | 6.30 | 5.83 |
| 2001 | 36034865.37 | 66747996.90 | 96266843.10 | 10159426.1 | 176214 | 390275 | 314560 | 29585 | 204.49 | 171.03 | 306.03 | 343.397 | 8.25 | 11.57 | 6.42 | 5.87 |
| 2002 | 36011985.12 | 66664264.05 | 96220539.36 | 10158586.6 | 177750 | 394769 | 316515 | 29624 | 202.56 | 168.86 | 304 | 342.91 | 8.38 | 11.84 | 6.55 | 5.92 |
| 2003 | 35993134.53 | 66589604.46 | 96172232.49 | 10156551.2 | 179261 | 398553 | 319181 | 29766 | 200.78 | 167.07 | 301.3 | 341.21 | 8.49 | 12.07 | 6.67 | 6.00 |
| 2004 | 35958794.76 | 66455005.5 | 96103743.48 | 10154953 | 181516 | 406793 | 323359 | 29858 | 198.1 | 163.36 | 297.2 | 340.108 | 8.67 | 12.41 | 6.82 | 6.06 |
| 2005 | 35931566.61 | 66324192.66 | 96057475.29 | 10154059.5 | 183509 | 417033 | 326285 | 29919 | 195.8 | 159.03 | 294.39 | 339.384 | 8.80 | 12.74 | 6.95 | 6.10 |
| 2006 | 35912585.43 | 66212460 | 96010221.51 | 10152790.2 | 185147 | 425459 | 329592 | 29950 | 193.97 | 155.62 | 291.3 | 338.99 | 8.92 | 13.06 | 7.09 | 6.16 |
| 2007 | 35896558.41 | 66107432.52 | 95951400.93 | 10150962.9 | 186470 | 433345 | 334607 | 30050 | 192.5 | 152.55 | 286.76 | 337.8 | 9.01 | 13.36 | 7.30 | 6.24 |
| 2008 | 35883475.92 | 65988001.89 | 95895877.14 | 10149982.6 | 187662 | 444233 | 338884 | 30095 | 191.21 | 148.54 | 282.97 | 337.26 | 9.09 | 13.67 | 7.47 | 6.29 |
| 2009 | 35875762.83 | 65910057.21 | 95865427.26 | 10148006.2 | 188274 | 451217 | 341003 | 30251 | 190.55 | 146.07 | 281.13 | 335.46 | 9.16 | 13.93 | 7.58 | 6.38 |
| 2010 | 35857374.39 | 65820677.58 | 95762364.57 | 10144063.3 | 189760 | 460043 | 352962 | 30477 | 188.96 | 143.07 | 271.31 | 332.84 | 9.30 | 14.21 | 7.93 | 6.58 |
| 2011 | 35845790.49 | 65742682.23 | 95700789.36 | 10140412.5 | 190861 | 469644 | 360263 | 30836 | 187.81 | 139.98 | 265.64 | 328.84 | 9.40 | 14.46 | 8.14 | 6.74 |
| 2012 | 35827294.77 | 65637624.15 | 95608528.11 | 10135846.3 | 192761 | 482576 | 372318 | 31186 | 185.86 | 136.01 | 256.79 | 325.01 | 9.63 | 14.89 | 8.46 | 6.94 |
| 2013 | 35818961.76 | 65546771.67 | 95566329.90 | 10132916.8 | 193882 | 492866 | 375950 | 31464 | 184.75 | 132.99 | 254.19 | 322.04 | 9.73 | 15.31 | 8.63 | 7.07 |
| 2014 | 35804993.22 | 65410002.36 | 95513173.92 | 10130383.7 | 195730 | 511591 | 380439 | 31641 | 182.93 | 127.86 | 251.06 | 320.16 | 9.86 | 15.87 | 8.85 | 7.19 |
| 2015 | 35793252.72 | 65283006.69 | 95461849.98 | 10128884.6 | 197289 | 527821 | 386079 | 31784 | 181.42 | 123.68 | 247.26 | 318.678 | 9.92 | 16.27 | 9.04 | 7.26 |
| 2016 | 35713407.15 | 64946605.59 | 95057634.51 | 10104777.9 | 208621 | 564359 | 429106 | 33953 | 171.19 | 115.08 | 221.52 | 297.61 | 10.35 | 17.39 | 10.01 | 8.03 |
| 2017 | 35664308.10 | 64692068.13 | 94714888.68 | 10088566.8 | 213271 | 587989 | 460817 | 34960 | 167.22 | 110.02 | 205.53 | 288.57 | 10.62 | 18.00 | 10.70 | 8.36 |
| sig. Level | *** | *** | *** | *** | *** | *** | *** | *** | *** | *** | *** | *** | *** | *** | *** | *** |
| *z*-value | -5.76 | -5.76 | -5.76 | -5.76 | 5.76 | 5.76 | 5.76 | 5.76 | -5.76 | -5.76 | -5.76 | -5.15 | 5.76 | 5.76 | 5.76 | 5.76 |

Significance: ***<0.001

**Table S9**. Changes in the forest cover area, number of patches, mean patch size, and edge density in primary forest (PF) and non-primary forest (NPF) from 2000 to 2017 in the Brazilian Legal Amazon (BLA). The significance of the trend was estimated by using the Mann–Kendall test.

| **Year** | **Forest cover area (ha)** | | **Number of patches** | | **Mean patch size (ha)** | | **Edge density (m/ha)** | |
| --- | --- | --- | --- | --- | --- | --- | --- | --- |
|  | PF | NPF | PF | NPF | PF | NPF | PF | NPF |
| 2000 | 329563072.6 | 77554832 | 565963 | 6212979 | 582.30 | 12.48 | 9.83 | 125.79 |
| 2001 | 328519325.7 | 76649768 | 648164 | 6245870 | 506.84 | 12.27 | 10.24 | 127.00 |
| 2002 | 326890027.8 | 75604385 | 767438 | 6333123 | 425.94 | 11.94 | 10.79 | 128.74 |
| 2003 | 325333973.1 | 74718792 | 870279 | 6404124 | 373.82 | 11.66 | 11.31 | 130.23 |
| 2004 | 323306974.7 | 73726564 | 1010427 | 6486158 | 319.97 | 11.37 | 11.95 | 131.99 |
| 2005 | 321446669 | 72861723 | 1170576 | 6602563 | 274.60 | 11.04 | 12.51 | 133.72 |
| 2006 | 320023049.3 | 72199039 | 1298088 | 6699229 | 246.53 | 10.78 | 12.98 | 135.37 |
| 2007 | 318847411.1 | 71582302 | 1409630 | 6784122 | 226.19 | 10.55 | 13.45 | 137.13 |
| 2008 | 317755379.7 | 71020077 | 1519023 | 6859167 | 209.18 | 10.35 | 13.82 | 138.65 |
| 2009 | 317049742.8 | 70566186 | 1592742 | 6930337 | 199.05 | 10.18 | 14.10 | 140.09 |
| 2010 | 315884557.8 | 69934201 | 1730274 | 7003008 | 182.56 | 9.99 | 14.58 | 141.94 |
| 2011 | 315082391.4 | 69471392 | 1831521 | 7066744 | 172.03 | 9.83 | 14.88 | 143.33 |
| 2012 | 313956459.1 | 68798500 | 1988639 | 7179260 | 157.87 | 9.58 | 15.37 | 145.93 |
| 2013 | 313266129.4 | 68233793 | 2097610 | 7303105 | 149.34 | 9.34 | 15.82 | 148.79 |
| 2014 | 312234744.1 | 67489118 | 2251455 | 7482644 | 138.68 | 9.02 | 16.35 | 152.08 |
| 2015 | 311332163.9 | 66831899 | 2390386 | 7649415 | 130.24 | 8.74 | 16.78 | 154.67 |
| 2016 | 308404077.5 | 65568844 | 2771542 | 7914906 | 111.27 | 8.28 | 18.26 | 159.44 |
| 2017 | 306135698.8 | 64365584 | 2999287 | 8157584 | 102.06 | 7.89 | 19.01 | 163.64 |
| sig. level | *** | *** | *** | *** | *** | *** | *** | *** |
| *z*-value | -5.76 | -5.76 | 5.76 | 5.76 | -5.76 | -5.76 | 5.76 | 5.76 |

Significance: ***<0.001

**Table S10***.* Changes in the number of forest cover fragments in primary forest (PF) and non-primary forest (NPF) areas in each size category from 2000 to 2017 within the Brazilian Legal Amazon (BLA). The significance of the trend was estimated by using the Mann–Kendall test.

|  | **Number of forest cover fragments** | | | | | | | | | | | | | | | |
| --- | --- | --- | --- | --- | --- | --- | --- | --- | --- | --- | --- | --- | --- | --- | --- | --- |
| Year | **<1 ha** | | **≥1 and <6.25 ha** | | **≥6.25 and <50 ha** | | **≥50 and <100 ha** | | **≥100 and <200 ha** | | **≥200 and <500 ha** | | **≥500 and <1000 ha** | | **≥1000 ha** | |
|  | PF | NPF | PF | NPF | PF | NPF | PF | NPF | PF | NPF | PF | NPF | PF | NPF | PF | NPF |
| 2000 | 413730 | 4763244 | 78046 | 914984 | 48320 | 427025 | 9215 | 51100 | 6209 | 28240 | 5241 | 17240 | 2211 | 5750 | 2991 | 5396 |
| 2001 | 484844 | 4778091 | 86516 | 927650 | 50518 | 432888 | 9409 | 51213 | 6348 | 28081 | 5293 | 16929 | 2241 | 5683 | 2995 | 5335 |
| 2002 | 585990 | 4844109 | 100304 | 944819 | 54226 | 437733 | 9761 | 51207 | 6486 | 27787 | 5351 | 16574 | 2270 | 5646 | 3050 | 5248 |
| 2003 | 672595 | 4900491 | 112522 | 957842 | 57685 | 440175 | 10044 | 51089 | 6588 | 27315 | 5446 | 16401 | 2292 | 5585 | 3107 | 5226 |
| 2004 | 791002 | 4968870 | 128792 | 971064 | 62263 | 441403 | 10477 | 50744 | 6791 | 27076 | 5528 | 16278 | 2382 | 5549 | 3192 | 5174 |
| 2005 | 927326 | 5071141 | 146770 | 986077 | 67072 | 441533 | 10885 | 50305 | 7103 | 26760 | 5688 | 16116 | 2479 | 5516 | 3253 | 5115 |
| 2006 | 1036646 | 5158700 | 160483 | 995810 | 70784 | 441503 | 11190 | 50032 | 7358 | 26586 | 5791 | 16038 | 2530 | 5472 | 3306 | 5088 |
| 2007 | 1132269 | 5235677 | 172775 | 1003480 | 73796 | 441905 | 11462 | 49934 | 7509 | 26548 | 5929 | 16068 | 2540 | 5458 | 3350 | 5052 |
| 2008 | 1228333 | 5305495 | 183164 | 1008976 | 76243 | 441876 | 11639 | 49799 | 7626 | 26489 | 6040 | 16057 | 2575 | 5429 | 3403 | 5046 |
| 2009 | 1293980 | 5370955 | 189397 | 1014244 | 77717 | 442358 | 11817 | 49754 | 7690 | 26506 | 6114 | 16090 | 2597 | 5403 | 3430 | 5027 |
| 2010 | 1419211 | 5441614 | 199041 | 1016885 | 79769 | 441833 | 12029 | 49660 | 7830 | 26520 | 6266 | 16107 | 2654 | 5394 | 3474 | 4995 |
| 2011 | 1513629 | 5502523 | 204458 | 1019980 | 80760 | 441799 | 12233 | 49612 | 7938 | 26423 | 6320 | 16064 | 2682 | 5362 | 3501 | 4981 |
| 2012 | 1658836 | 5609341 | 214261 | 1026008 | 82380 | 441920 | 12440 | 49380 | 8059 | 26387 | 6405 | 15934 | 2716 | 5342 | 3542 | 4948 |
| 2013 | 1756279 | 5721159 | 223656 | 1036616 | 84212 | 443498 | 12633 | 49349 | 8132 | 26313 | 6439 | 15929 | 2733 | 5306 | 3526 | 4935 |
| 2014 | 1894610 | 5883605 | 236333 | 1051629 | 86643 | 445807 | 12794 | 49297 | 8259 | 26345 | 6512 | 15826 | 2770 | 5245 | 3534 | 4890 |
| 2015 | 2018480 | 6033502 | 248486 | 1066022 | 89197 | 448423 | 12935 | 49340 | 8420 | 26320 | 6531 | 15741 | 2798 | 5231 | 3539 | 4836 |
| 2016 | 2346112 | 6266333 | 290383 | 1094437 | 99269 | 452975 | 13630 | 49409 | 8838 | 26236 | 6816 | 15570 | 2895 | 5168 | 3599 | 4778 |
| 2017 | 2538618 | 6475401 | 317692 | 1123185 | 106155 | 458151 | 14152 | 49454 | 9118 | 26122 | 6980 | 15494 | 2897 | 5086 | 3675 | 4691 |
| sig. level | *** | *** | *** | *** | *** | *** | *** | *** | *** | *** | *** | *** | *** | *** | *** | *** |
| *z*-value | 5.56 | 5.56 | 5.56 | 5.56 | 5.56 | 4.74 | 5.56 | -4.98 | 5.56 | -5.15 | 5.56 | -4.65 | 5.56 | -5.56 | 5.31 | -5.56 |

Significance: ***<0.001
